# Machine learning links unresolving secondary pneumonia to mortality in patients with severe pneumonia, including COVID-19

**DOI:** 10.1172/JCI170682

**Published:** 2023-06-15

**Authors:** Catherine A. Gao, Nikolay S. Markov, Thomas Stoeger, Anna Pawlowski, Mengjia Kang, Prasanth Nannapaneni, Rogan A. Grant, Chiagozie Pickens, James M. Walter, Jacqueline M. Kruser, Luke Rasmussen, Daniel Schneider, Justin Starren, Helen K. Donnelly, Alvaro Donayre, Yuan Luo, G.R. Scott Budinger, Richard G. Wunderink, Alexander V. Misharin, Benjamin D. Singer, The NU SCRIPT Study Investigators

**Affiliations:** 1 Division of Pulmonary and Critical Care Medicine, Department of Medicine, Northwestern University Feinberg School of Medicine, Chicago, Illinois, USA.; 2 Department of Chemical and Biological Engineering, Northwestern University, McCormick School of Engineering, Evanston, Illinois, USA.; 3 Northwestern Medicine Enterprise Data Warehouse, Northwestern University Feinberg School of Medicine, Chicago, Illinois, USA.; 4 Division of Allergy, Pulmonary and Critical Care, Department of Medicine, University of Wisconsin School of Medicine and Public Health, Madison, Wisconsin, USA.; 5 Division of Health and Biomedical Informatics, Department of Preventive Medicine and; 6 Simpson Querrey Lung Institute for Translational Science (SQLIFTS), Northwestern University Feinberg School of Medicine, Chicago, Illinois, USA.; 7 The Northwestern University (NU) Successful Clinical Response In Pneumonia Therapy (SCRIPT) Study Investigators are listed in Supplemental Table 3.

**Keywords:** Infectious disease, Pulmonology, Bacterial infections, Bioinformatics

## Abstract

**BACKGROUND:**

Despite guidelines promoting the prevention and aggressive treatment of ventilator-associated pneumonia (VAP), the importance of VAP as a driver of outcomes in mechanically ventilated patients, including patients with severe COVID-19, remains unclear. We aimed to determine the contribution of unsuccessful treatment of VAP to mortality for patients with severe pneumonia.

**METHODS:**

We performed a single-center, prospective cohort study of 585 mechanically ventilated patients with severe pneumonia and respiratory failure, 190 of whom had COVID-19, who underwent at least 1 bronchoalveolar lavage. A panel of intensive care unit (ICU) physicians adjudicated the pneumonia episodes and endpoints on the basis of clinical and microbiological data. Given the relatively long ICU length of stay (LOS) among patients with COVID-19, we developed a machine-learning approach called *CarpeDiem*, which grouped similar ICU patient-days into clinical states based on electronic health record data.

**RESULTS:**

*CarpeDiem* revealed that the long ICU LOS among patients with COVID-19 was attributable to long stays in clinical states characterized primarily by respiratory failure. While VAP was not associated with mortality overall, the mortality rate was higher for patients with 1 episode of unsuccessfully treated VAP compared with those with successfully treated VAP (76.4% versus 17.6%, *P* < 0.001). For all patients, including those with COVID-19, *CarpeDiem* demonstrated that unresolving VAP was associated with a transitions to clinical states associated with higher mortality.

**CONCLUSIONS:**

Unsuccessful treatment of VAP is associated with higher mortality. The relatively long LOS for patients with COVID-19 was primarily due to prolonged respiratory failure, placing them at higher risk of VAP.

**FUNDING:**

National Institute of Allergy and Infectious Diseases (NIAID), NIH grant U19AI135964; National Heart, Lung, and Blood Institute (NHLBI), NIH grants R01HL147575, R01HL149883, R01HL153122, R01HL153312, R01HL154686, R01HL158139, P01HL071643, and P01HL154998; National Heart, Lung, and Blood Institute (NHLBI), NIH training grants T32HL076139 and F32HL162377; National Institute on Aging (NIA), NIH grants K99AG068544, R21AG075423, and P01AG049665; National Library of Medicine (NLM), NIH grant R01LM013337; National Center for Advancing Translational Sciences (NCATS), NIH grant U01TR003528; Veterans Affairs grant I01CX001777; Chicago Biomedical Consortium grant; Northwestern University Dixon Translational Science Award; Simpson Querrey Lung Institute for Translational Science (SQLIFTS); Canning Thoracic Institute of Northwestern Medicine.

## Graphical abstract

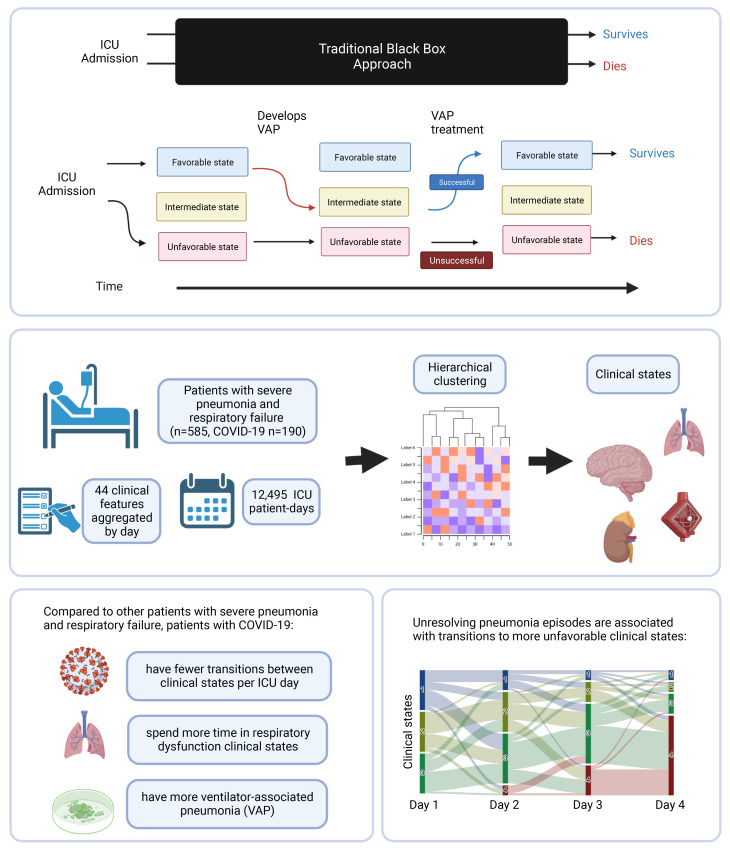

## Introduction

Several groups, including ours, have reported that the duration of intensive care unit (ICU) stays and mechanical ventilation are more than twice as long for patients with SARS-CoV-2 pneumonia compared with patients with respiratory failure complicating pneumonia due to other pathogens and patients with other causes of acute respiratory distress syndrome (ARDS) (1–15). Based on analyses of peripheral blood samples from patients with severe versus mild COVID-19, some investigators have hypothesized that the long ICU length of stay (LOS) among patients with SARS-CoV-2 pneumonia is secondary to multiple organ dysfunction (reviewed in ref. 16). This hypothesis poorly explains the parallel observation that, despite their longer LOS, mortality is similar for patients with severe SARS-CoV-2 pneumonia compared with patients with pneumonia and respiratory failure secondary to other etiologies (2–4, 6). Intercurrent ICU events that disproportionately affect patients with COVID-19 might explain the disconnect between ICU LOS and mortality.

In a review of autopsy samples stored from the 1918 influenza A pandemic, Fauci and colleagues suggested an unexpectedly important contribution of secondary bacterial infection to mortality after severe viral pneumonia (17). Recent data suggest that secondary pneumonia is present in up to 40% and pneumonia or diffuse alveolar damage in over 90% of autopsy specimens obtained from patients with acute SARS-CoV-2 infection (18). Consistent with these observations, we and others found high rates of ventilator-associated pneumonia (VAP) in patients with SARS-CoV-2 pneumonia requiring mechanical ventilation, suggesting that bacterial superinfections such as VAP may contribute to mortality in patients with COVID-19 (7, 19–22). These findings prompted an alternative hypothesis that a relatively low mortality rate directly attributable to primary SARS-CoV-2 infection is offset by a greater risk of death attributable to unresolving VAP (23).

Testing the hypothesis that unresolving VAP explains the disconnect between ICU LOS and mortality for patients with severe SARS-CoV-2 pneumonia poses 2 challenges. First, traditional methods to compare ICU outcomes standardize the severity of illness on ICU admission and treat the entirety of the ensuing ICU stay as a single event (24–26). This approach fails to capture ICU complications, such as VAP, that by definition are rarely present on ICU admission but likely alter the trajectory of the patient toward unfavorable outcomes, particularly when the ICU LOS is long. Indeed, few ICU studies have attempted to examine the effect of late ICU interventions and complications on patient outcomes (27–29). Second, if they resolve, VAP episodes may contribute to prolonged ICU LOS, without worsening the outcome. Nevertheless, most studies use insensitive methods to diagnose VAP and measure the response to therapy (30).

We analyzed the contribution of VAP to mortality in 585 patients with severe pneumonia and respiratory failure, including 190 patients with severe SARS-CoV-2 pneumonia, who were enrolled in the Successful Clinical Response in Pneumonia Therapy (SCRIPT) study. All patients underwent bronchoalveolar lavage (BAL) sampling paired with comprehensive microbiological diagnostics at the time of study enrollment and whenever pneumonia was clinically suspected over the course of their intubation. Clinicians performed BAL sampling as part of routine clinical care and used BAL fluid studies to guide antimicrobial therapy (7). To disentangle the effect of VAP on outcomes over the course of the ICU stay, we developed a machine-learning approach we termed *CarpeDiem*, which clustered individual patient-days in the ICU using clinical parameters extracted from the electronic health record (EHR). Because key clinical data fed the *CarpeDiem* algorithm, these clusters represented clinical states that were differentially associated with hospital mortality. The *CarpeDiem* framework allowed us to examine transitions between clinical states associated with favorable (lower mortality) or unfavorable (higher mortality) outcomes. Indeed, *CarpeDiem* revealed that the long ICU LOS among patients with COVID-19 relative to patients with pneumonia secondary to other pathogens resulted from excess days in clinical states characterized by severe hypoxemic respiratory failure with significantly fewer transitions between states when normalized for their longer LOS. Unresolving episodes of VAP were associated with transitions to clinical states associated with greater mortality. These data suggest that mortality associated with severe SARS-CoV-2 pneumonia is more often associated with respiratory failure that increases the risk of unresolving VAP and is less frequently associated with multiple organ dysfunction.

## Results

### Demographics.

**Figure 1:**
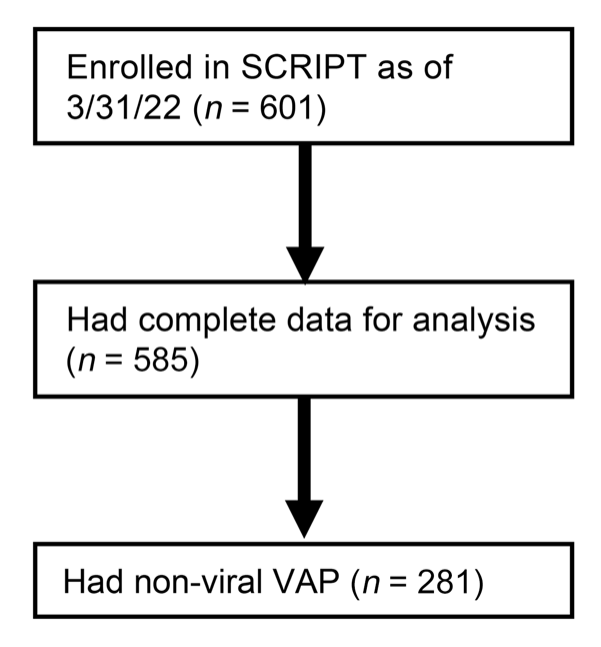
CONSORT diagram of the SCRIPT study participants and analysis.

**Figure 2:**
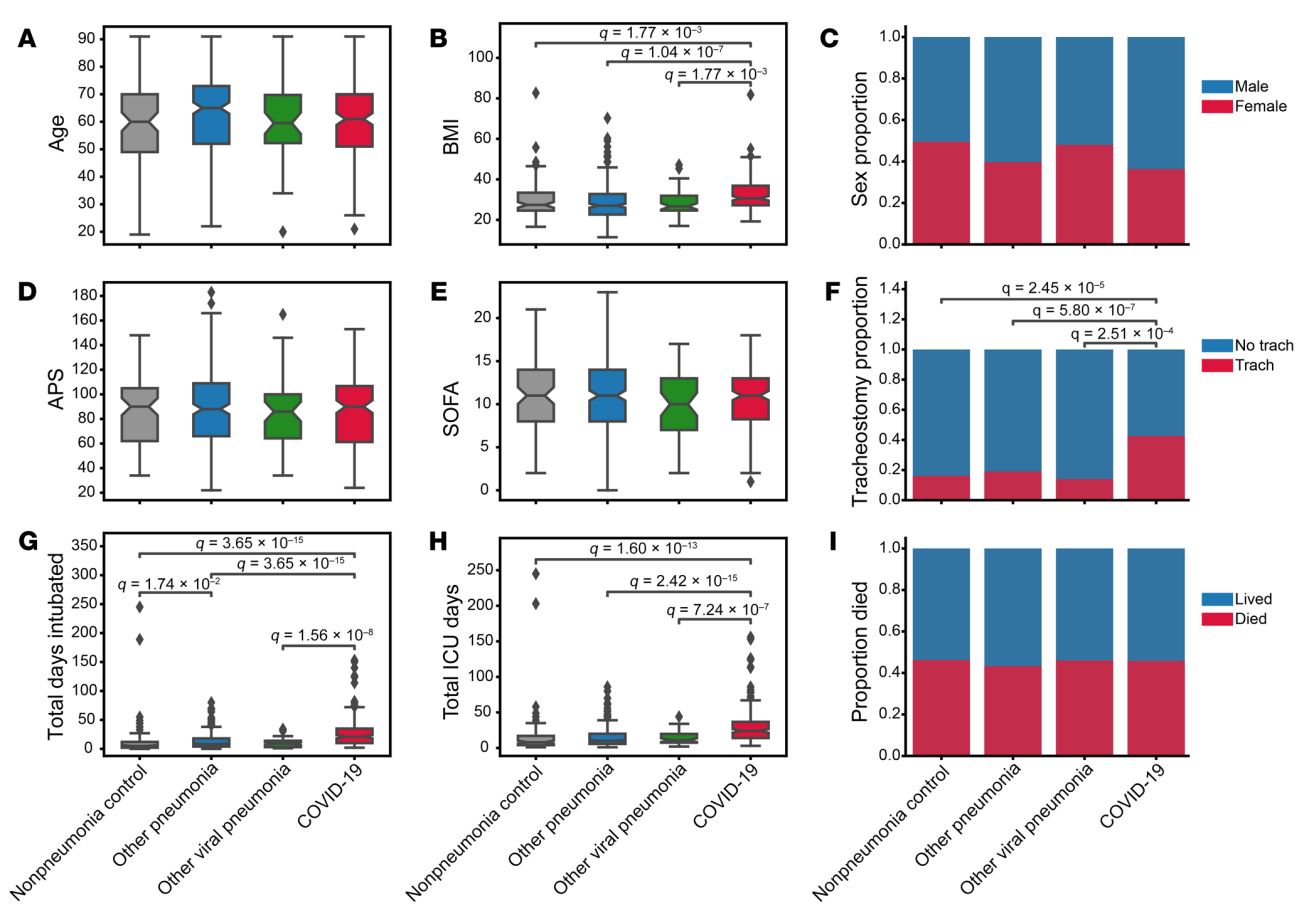
Demographics and outcomes of the cohort grouped by pneumonia category. Distribution of (**A**) patient age in years, (**B**) BMI in kg/m^2^ (1 patient did not have BMI data available), (**C**) sex, (**D**) APS, (**E**) SOFA score, (**F**) tracheostomy placement, (**G**) duration of intubation, (**H**) length of ICU stay, and (**I**) hospital mortality. Total days intubated (**G**) and total ICU days (**H**) include only days at our hospital and do not capture intubation duration or ICU LOS at a transferring hospital. Data on patients who lived include dispositions of discharge to home, acute inpatient rehabilitation, and admission to a long-term acute care hospital (LTACH) or skilled nursing facility (SNF) (see Supplemental Table 1). Data on patients who died include patients who died in the hospital, patients who underwent lung transplantation for refractory respiratory failure, and patients who were transferred to home or inpatient hospice. The APS score from APACHE IV was calculated from the worst value within the first 2 ICU days, and SOFA score was calculated from the worst value within the first 2 ICU days. In the box-and-whisker plots, the box shows quartiles and the median, and the whiskers show the minimum and maximum values except for outliers, which are shown as individual data points. Notches are bootstrapped 95% CI of the median. Numerical values were compared with the Mann-Whitney *U* test with FDR correction using the Benjamini-Hochberg procedure. Categorical values were compared using Fisher’s exact tests with FDR correction using the Benjamini-Hochberg procedure. A *q* value of less than 0.05 was the threshold for statistical significance. Numerical values and additional details are available in Supplemental Tables 1 and 2.

Of 601 patients enrolled in SCRIPT between June 2018 and March 2022, 585 had an adjudicated pneumonia category and clinical endpoints at the time of analysis (Figure 1): 190 had COVID-19, 50 had pneumonia secondary to other respiratory viruses, 252 had other pneumonia (bacterial), and 93 were initially suspected of having pneumonia yet were subsequently adjudicated as having respiratory failure unrelated to pneumonia (nonpneumonia controls). Except for BMI, demographics such as age and sex were similar between the groups (Figure 2, A–C, and Supplemental Table 1, which also includes a description of patient comorbidities). Severity of illness, as measured by the Acute Physiology Score (APS) from Acute Physiology And Chronic Health Evaluation (APACHE) IV (31) and the Sequential Organ Failure Assessment (SOFA) score (24, 32) in the first 2 days of admission, did not differ between the groups (Figure 2, D and E). Patients across the pneumonia categories underwent intubation following a similar duration of time in the ICU, with a trend toward later intubation in patients with COVID-19 (Supplemental Figure 1A and Supplemental Table 2; supplemental material available online with this article; https://doi.org/10.1172/JCI170682DS1). At the time of intubation, the SOFA scores were similar for patients with COVID-19 compared with other patients in the cohort who were intubated after admission to our hospital (Supplemental Table 2). On the first day of intubation, patients with COVID-19 had lower oxygen saturation levels despite a higher fraction of inspired O_2_ (FiO_2_ ) (Supplemental Table 2). They required higher levels of positive end-expiratory pressure (PEEP) but had lower heart rates and were receiving lower doses of norepinephrine (Supplemental Table 2). Despite a similar overall severity of illness on ICU admission, the durations of intubation and ICU stays were more than twice as long among patients with SARS-CoV-2 pneumonia compared with any other group, reflected by a higher frequency of tracheostomy and longer ICU LOS (Figure 2, F–H, and Supplemental Figure 2). The longer ICU LOS persisted when patients who received extracorporeal membrane oxygenation (ECMO) support or who were received in external transfer (31.4% of the cohort) were excluded from the cohort (Supplemental Figure 1, B and C). Hospital mortality did not differ between groups (Figure 2I). A similar fraction of patients with SARS-CoV-2 pneumonia received corticosteroids during their ICU stay compared with the rest of the cohort, but patients with SARS-CoV-2 pneumonia received higher cumulative doses (Supplemental Table 1). Patients with SARS-CoV-2 pneumonia were more likely to receive IL-6 receptor antagonists and remdesivir (Supplemental Table 1).

### CarpeDiem: a machine-learning approach to time-series data in the ICU.

**Figure 3:**
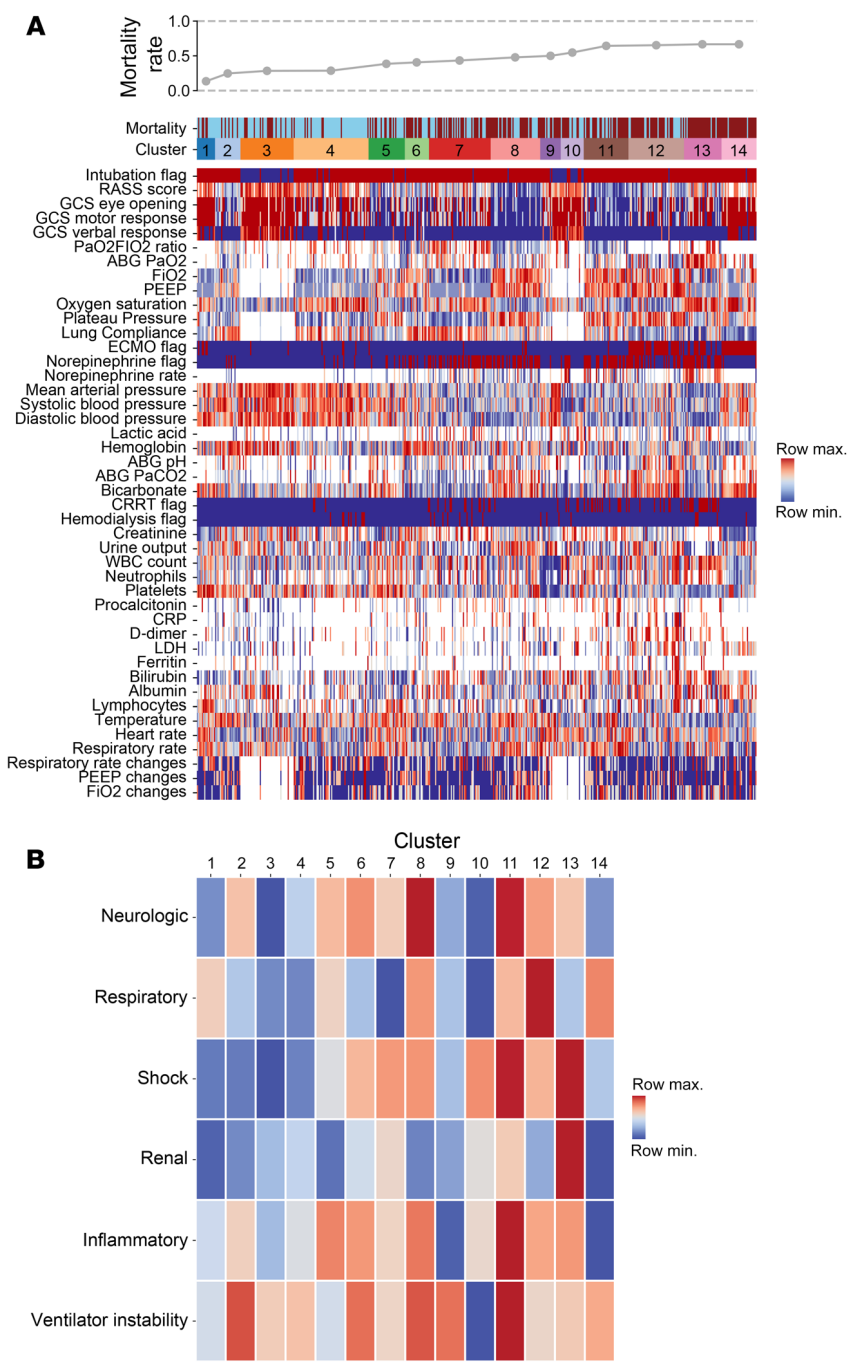
*CarpeDiem* groups patient-days into clusters representing clinical states associated with differential hospital mortality. (**A**) Heatmap of 44 clinical parameters with columns (representing 12,495 ICU patient-days for 585 patients) grouped into *CarpeDiem*-generated clusters (clinical states) ordered from the lowest to highest mortality rates. Rows are sorted into physiologically related groups. The top row signifies the hospital mortality outcome of the patient shown in the column (blue = lived, red = died). The hospital mortality rate associated with each cluster is shown above the heatmap. (**B**) Heatmap of the composite signal from each cluster and physiological group with ordering the same as in **A**.

To address the challenge of comparing intercurrent ICU events between groups with different ICU LOS, we developed a machine-learning approach, *CarpeDiem*, to discretize each patient-day in the ICU. For all 12,495 ICU patient-days for the cohort, we extracted clinical data from the EHR describing 44 key clinical parameters, including flags for organ failures requiring mechanical support (e.g., mechanical ventilation, renal replacement therapy, and ECMO), continuously recorded clinical parameters (e.g., vital signs and doses of norepinephrine), and commonly measured laboratory values (Supplemental Figure 3A). Variables used to calculate the SOFA score are a subset of these parameters. Importantly, patient-intrinsic variables (e.g., demographics, BMI, tracheostomy, and diagnosis), biochemical and microbiological analyses of BAL fluid studies, and adjudication of VAP episodes were not included in the model. Correlation analysis identified expected associations between mathematically or physiologically coupled variables (e.g., plateau pressure, PEEP, and lung compliance; partial pressure of CO_2_ [PaCO_2_ and bicarbonate) and revealed clinically recognizable correlated features (e.g., ECMO, D-dimer, and lactate dehydrogenase [LDH]) (Supplemental Figure 3B). After reducing the weight of these highly correlated features, we performed clustering using several methods, all of which yielded similar results (Supplemental Figure 4, A–C). We designed a clustering strategy based on the similarity between patient-days (see details in Supplemental Methods) and selected the number of clusters by choosing a near-maximal difference in mortality between pairwise comparisons of clusters (Supplemental Figure 5A) while limiting the number of cluster breaks to those that were determined to be clinically meaningful by 4 ICU physicians (CAG, GRSB, RGW, BDS). To explore the stability of our clustering approach, we randomly excluded patients from our cohort and independently reclustered this subset. While the overall patterns of clustering were similar, the assignment of patient-days to specific clusters differed (Supplemental Figure 5B). We visualized the resulting 14 clusters using heatmaps (Figure 3, A and B) and uniform manifold approximation and projection (UMAP) plots (Supplemental Figure 6). Median SOFA scores for the days in each cluster are shown in Supplemental Figure 7. Every cluster contained patients and patient-days from each pneumonia category, including COVID-19 (Supplemental Figure 8, A and B). Thus, the clinical states defined by *CarpeDiem* are useful to compare patient-days within a given cohort but do not represent a priori states to which patient-days can be prospectively assigned.

**Figure 4:**
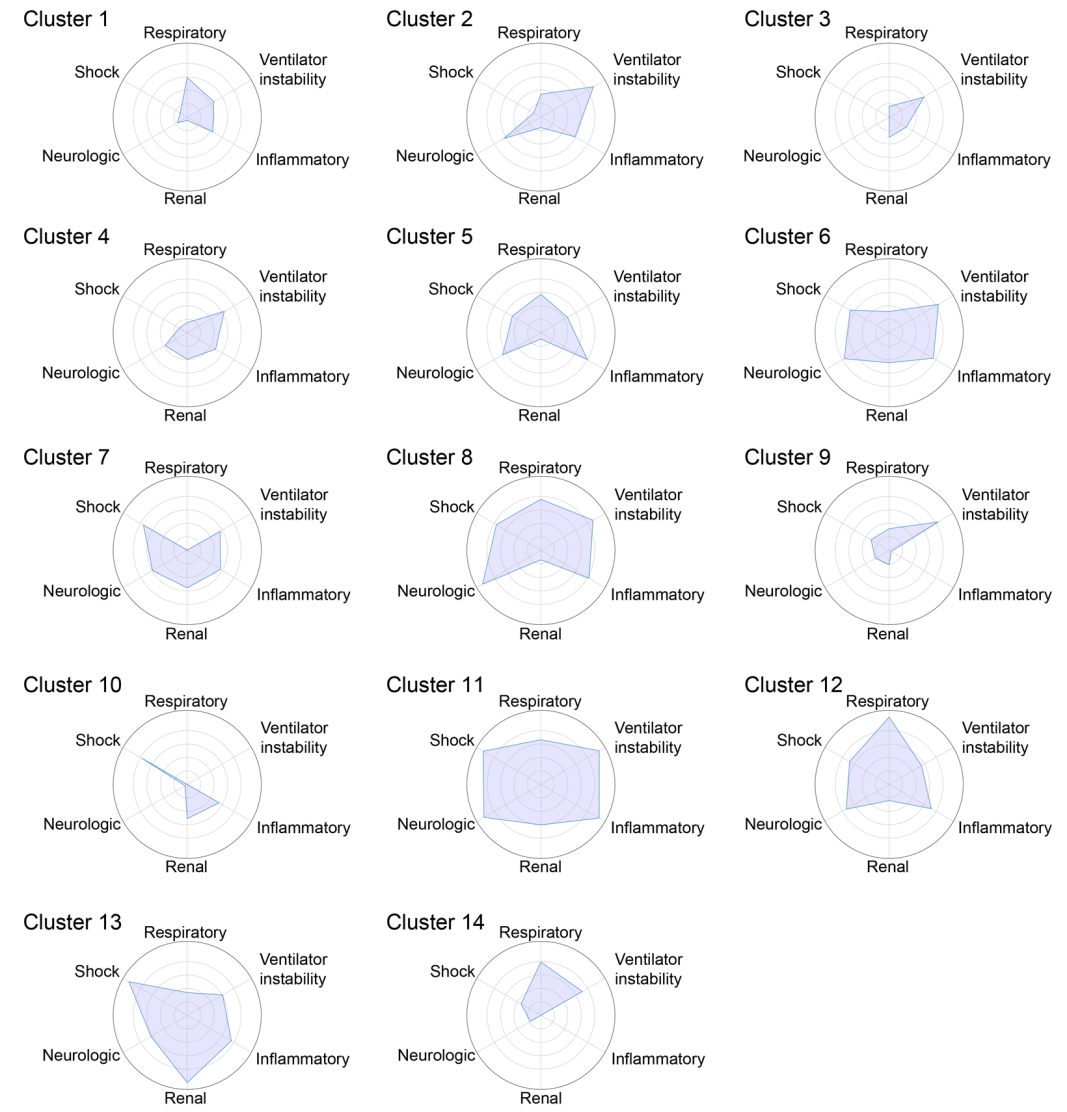
*CarpeDiem* clinical states have different patterns of organ dysfunction. Spider plots of minimum–maximum normalized composite features from Figure 3B for each clinical state. Circles indicate values of 0.2 (innermost), 0.4, 0.6, 0.8, and 1 (outermost).

As *CarpeDiem* uses physiological parameters and laboratory values evaluated by clinicians to develop a daily plan of care, the clusters generated by *CarpeDiem* are recognizable as clinical states. To visualize these data, we arranged the parameters into 6 physiological groups (neurologic, respiratory, shock, renal, inflammatory, and ventilator instability) and sorted the clusters in order of increasing mortality. The resulting heatmaps (Figure 3, A and B) and spider plots (Figure 4) revealed an association between patient-days characterized by multiple organ failure and mortality, findings consistent with published scoring systems (24–26). We compared mortality for each clinical state identified by *CarpeDiem* on the first, median, and last day in the ICU. On the first day of the ICU stay, only 2 of the clinical states were significantly associated with outcome (Supplemental Figure 9A). In contrast, the same analysis for the median and last ICU day for each patient revealed 8 and 9 significant associations, respectively, between clinical state and outcome (Supplemental Figure 9, B and C), supporting the construct validity of the *CarpeDiem*-generated clusters and the rationale to use *CarpeDiem* in an unsupervised fashion to evaluate all days of the ICU stay.

Critical care physicians (CAG, GRSB, RGW, BDS) used these visualizations to interpret the clinical states. For example, clinical state 12 represents patient-days with very severe respiratory failure (mostly days spent receiving ECMO support), moderately high levels of sedation, an intermediate level of shock without substantial renal failure, and relatively stable ventilator settings. Importantly, while enriched for patients receiving ECMO support, clinical state 12 consisted of days spanning the duration of the ICU stay (Supplemental Figure 10), supporting the notion that ECMO is a marker of persistent, severe respiratory failure rather than a salvage or perimortem intervention applied at the end of the ICU stay. An illustration of time-series data and transitions between clinical states over a selected patient’s ICU course is provided in Supplemental Figure 6J.

### Validation of the CarpeDiem approach in the MIMIC-IV data set.

We next determined whether the *CarpeDiem* approach could be used to analyze an external data set. Within the Medical Information Mart for Intensive Care IV (MIMIC-IV) database of ICU patients (33), we identified the subset of 1,284 ICU stays similar to those in our cohort. The *CarpeDiem* approach applied to 15,642 ICU patient-days using 27 clinical parameters, a subset of the 44 used above that were readily available in the MIMIC-IV database, identified 12 clusters (Supplemental Figure 11, A–C). Similar to our observations in the SCRIPT cohort, *CarpeDiem*-generated clusters in MIMIC-IV were clinically recognizable with increasing organ failure associated with mortality (Supplemental Figure 11, D and E). Although these results support the generalizability of the *CarpeDiem* approach, the clinical states observed in the MIMIC-IV cohort were not identical to those in the SCRIPT cohort. This observation might be expected, as, for example, MIMIC-IV had very few patients who received ECMO, underscoring the concept that clinical states cannot be assigned a priori in a given cohort.

### CarpeDiem reveals that the long LOS among patients with COVID-19 is associated with prolonged stays in clinical states characterized by severe respiratory failure.

**Figure 5:**
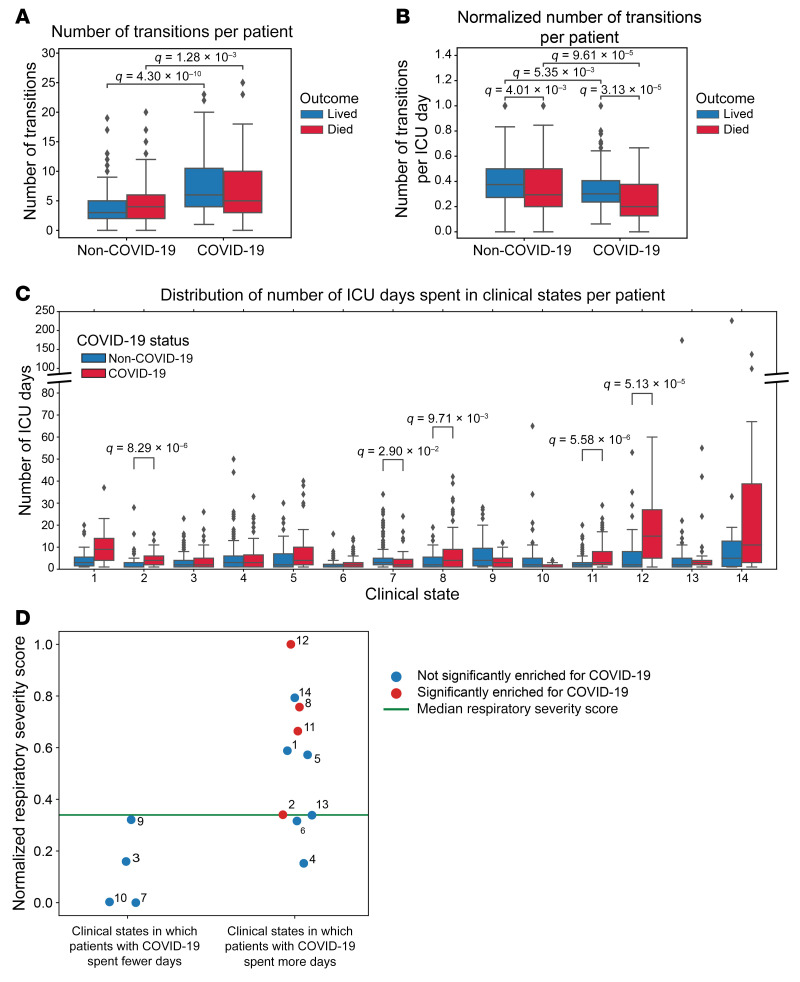
The long LOS among patients with COVID-19 is driven by a lower frequency of transitions, resulting in longer durations of time spent in certain clinical states. (**A**) Distribution of transitions per patient. (**B**) Distribution of transitions normalized by ICU LOS. (**C**) Distribution of ICU days spent in each clinical state per patient. The *y*-axis is discontinuous to accommodate all data points. (**D**) Respiratory severity score per clinical state, which is numbered next to each point, split by whether that cluster was enriched in patient-days for patients with COVID-19. Green line indicates the median respiratory severity score for the cohort. For the box-and-whisker plots, the box shows quartiles and the median, and whiskers show the minimum and maximum values except for outliers, which are shown as individual data points. Numerical values were compared using Mann-Whitney *U* tests with FDR correction using the Benjamini-Hochberg procedure. A *q* value of less than 0.05 was our threshold for statistical significance.

We reasoned that *CarpeDiem* could provide insight into the reasons why patients with severe SARS-CoV-2 pneumonia had longer ICU LOS relative to patients with pneumonia and respiratory failure secondary to other etiologies despite similar hospital mortality rates. We posited that this observation could result from (a) longer stays in a given clinical state with similar numbers of transitions between states, as would be observed for prolonged respiratory failure or (b) similar durations of stay in any given clinical state with a balanced increase in the number of transitions between favorable and unfavorable states, as might be observed in patients developing multiple organ dysfunction. Although the absolute number of transitions between clinical states was higher among patients with SARS-CoV-2 pneumonia when compared with all other patient groups in the cohort (Figure 5A and Supplemental Figure 12A), the frequency of transitions was significantly lower (Figure 5B and Supplemental Figure 12B). The longer ICU LOS experienced by patients with severe SARS-CoV-2 pneumonia resulted from significantly prolonged stays in 4 clinical states (Figure 5C). Clusters that were enriched in days from patients with COVID-19 had higher respiratory severity scores (Figure 3, Figure 4, and Figure 5D), illustrating that patients with COVID-19 spent a disproportionate amount of time in clusters characterized by hypoxemic respiratory failure. Time spent in clinical state 12, characterized by severe hypoxemic respiratory failure, accounted for 29.9% of the difference in ICU LOS experienced by patients with COVID-19. Overall, since some clusters were deficient in patients with COVID-19, time spent in the 4 clinical states that were significantly enriched in patients with COVID-19 accounted for over 100% of the difference in ICU LOS between patients with and without COVID-19.

To examine the robustness of our findings with regard to changes in the composition of the cohort, we randomly excluded 20% of the cohort and reclustered patient-days 500 times. As shown in Supplemental Figure 13, the main conclusions drawn from the full data set hold after random subsampling, including the finding that patients with COVID-19 experienced fewer transitions per day irrespective of outcome (as in Figure 5B) and experienced longer stays in clusters with high respiratory severity scores (as in Figure 5D).

To explore the potential utility of the *CarpeDiem* approach within the context of a randomized, controlled trial, we analyzed the 10 patients within SCRIPT who were also enrolled in a randomized, placebo-controlled trial of the IL-6 receptor antagonist sarilumab for the treatment of patients with respiratory failure secondary to COVID-19. The results of randomized, controlled trials of IL-6 receptor antagonists in patients with COVID-19 have been mixed (34), with some trials reporting benefit, while others, including this trial (35), did not. We calculated the sum of *CarpeDiem*-defined clinical state transitions occurring 3 and 5 days following randomization to sarilumab (*n* = 6) or placebo (*n* = 4). Even within this very small group, we observed significantly more favorable transitions in patients who received sarilumab compared with those who received placebo in the 3 days after drug administration (Supplemental Figure 14, A and B). In contrast, no statistically significant difference was evident 5 days after randomization (Supplemental Figure 14C).

### Unresolving VAP drives poor outcomes in patients with severe pneumonia, including pneumonia due to SARS-CoV-2.

**Figure 6:**
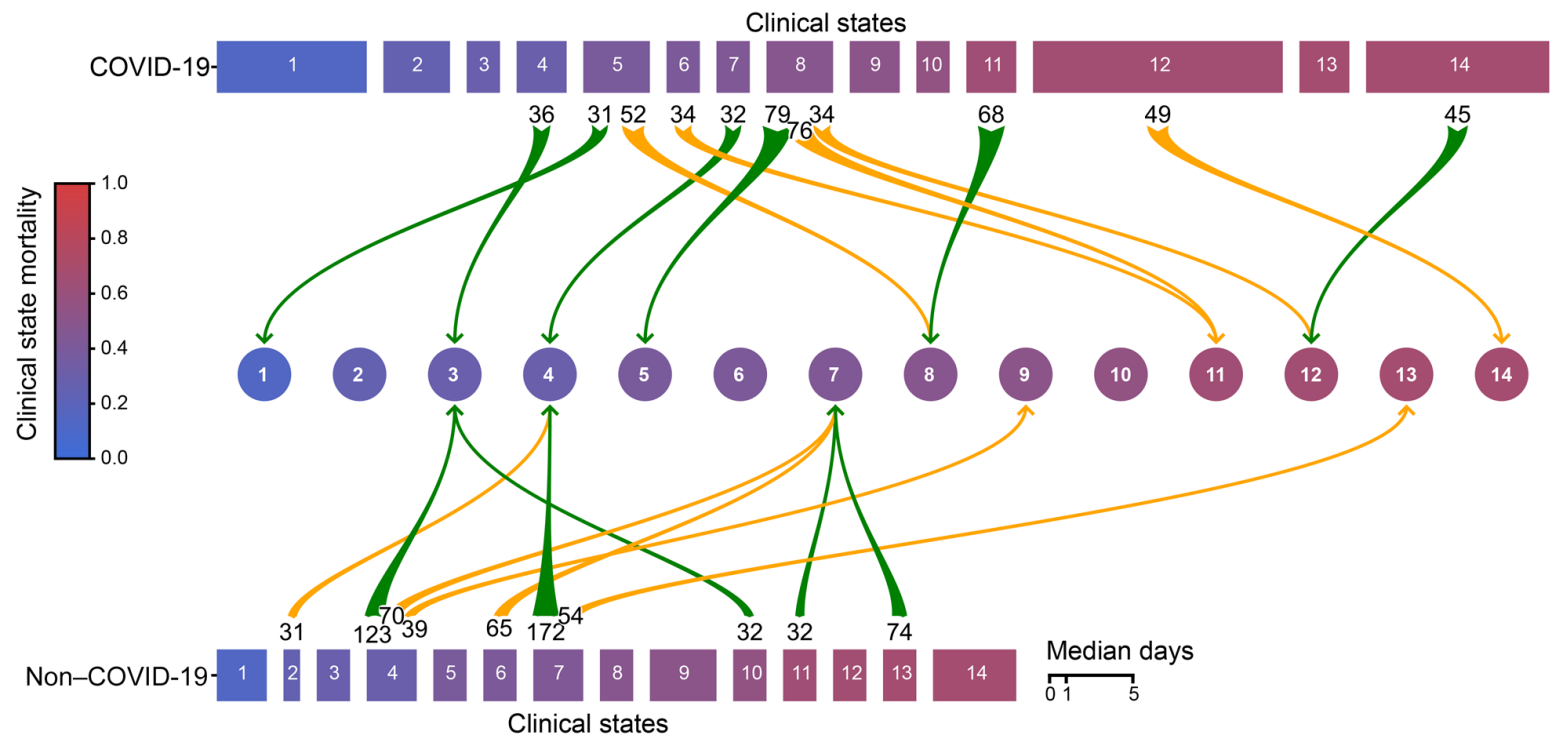
Patients with SARS-CoV-2 pneumonia have a longer LOS and fewer transitions between clinical states per day compared with patients with non–COVID-19–related respiratory failure. Clinical states are ordered and numbered 1–14 according to their associated mortality (blue to red). Rectangle width reflects the median number of days spent in each clinical state. Green arrows indicate transitions to a more favorable (lower mortality) clinical state; yellow arrows mark transitions to a less favorable (higher mortality) clinical state. Numbers at the arrow bases represent the number of transitions between the 2 clinical states connected by the arrow. Only transitions that occurred more than 30 times are shown.

Nearly all patients (97.4%) underwent transitions between clinical states over the course of their ICU stay (median [IQR] of 4[2,7] transitions per patient). We defined transitions as favorable if the mortality associated with the destination clinical state was lower than the originating state and vice versa. While the number of unfavorable transitions was similar in patients with SARS-CoV-2 pneumonia and other patients in the cohort, the number of favorable transitions was nominally lower in patients with SARS-CoV-2 pneumonia (Figure 6 and Supplemental Figure 15, A and B).

**Figure 7:**
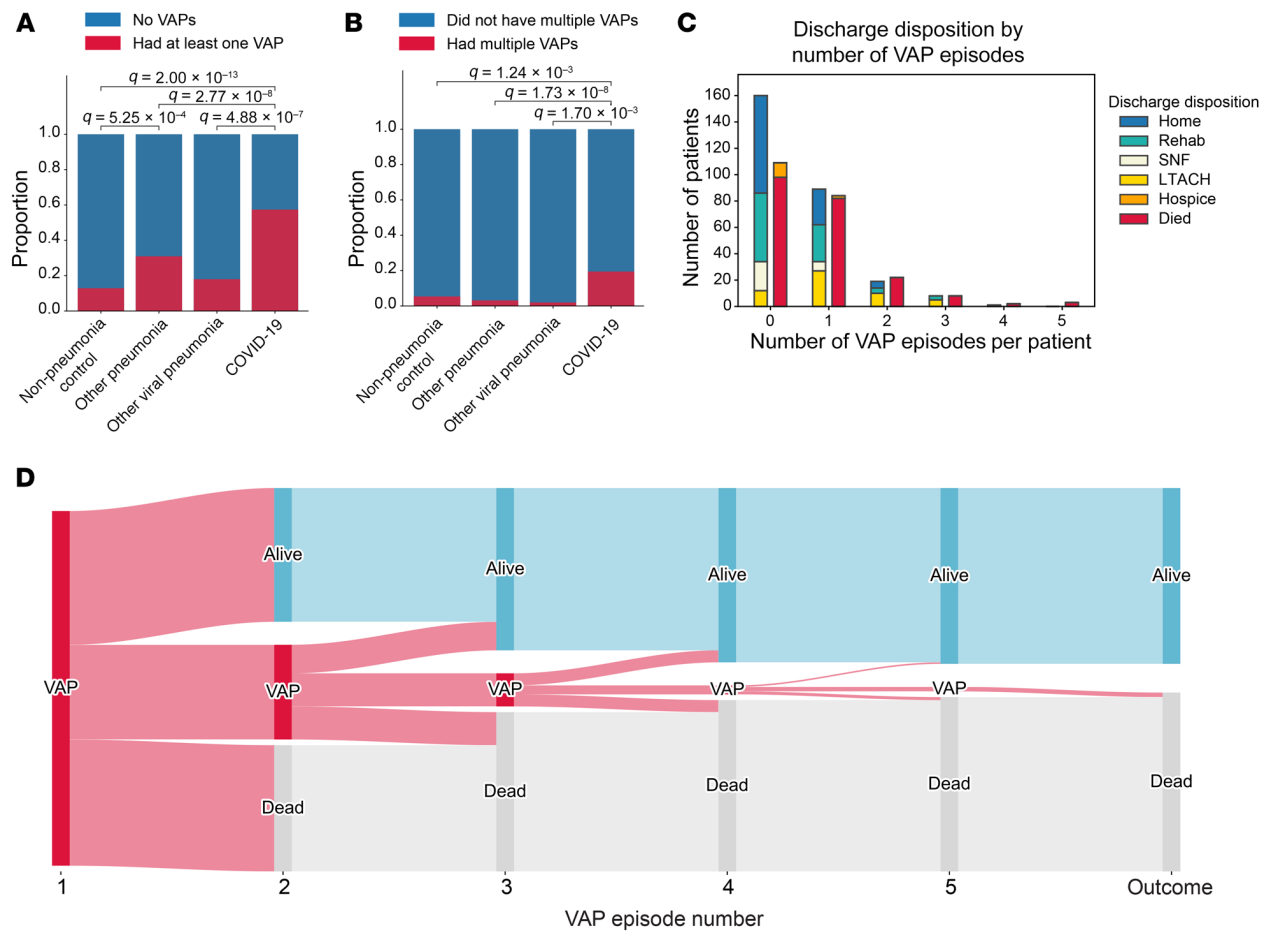
Patients with COVID-19 experience more VAP episodes than do patients without COVID-19. (**A**) Proportion of patients with at least 1 VAP. (**B**) Proportion of patients with more than 1 VAP. (**C**) Outcomes for patients experiencing different numbers of VAP episodes. Outcomes are displayed in 2 columns: the first column aggregates favorable discharge dispositions (home, rehabilitation, SNF, LTACH); the second column aggregates unfavorable discharge dispositions (hospice, died). (**D**) Sankey diagram of VAP episodes and outcomes for each VAP episode. Categorical values were compared using Fisher’s exact test with FDR correction using the Benjamini-Hochberg procedure. A *q* value of less than 0.05 was the threshold for statistical significance.

We hypothesized that VAP would, at least in part, explain the disconnect between ICU LOS and mortality in patients with COVID-19. Overall, 35.5% of patients in the cohort developed at least 1 episode of VAP during their ICU stay (25.0% among patients without COVID-19 compared with 57.4% among patients with COVID-19, *P* < 0.001) (Figure 7A). A total of 8.7% of patients in the cohort experienced more than 1 episode of VAP (3.5% among patients without COVID-19 compared with 19.5% among patients with COVID-19, *P* < 0.001) (Figure 7B). Mortality for patients with VAP has been reported to increase substantially with each ensuing episode, approaching 100% in patients with 3 or more episodes (36). In contrast, we found that the mortality rate associated with a single VAP episode did not differ from the mortality rate associated with multiple VAP episodes (48.6% with a single episode, 53.6% with 2 episodes, 50.0% with 3 episodes; *P* = NS) (Figure 7C), suggesting that a cure can be achieved even in patients with multiple VAP episodes. Nevertheless, the relatively small number of patients with multiple VAP episodes limited the power to detect small differences (Figure 7D).

**Figure 8:**
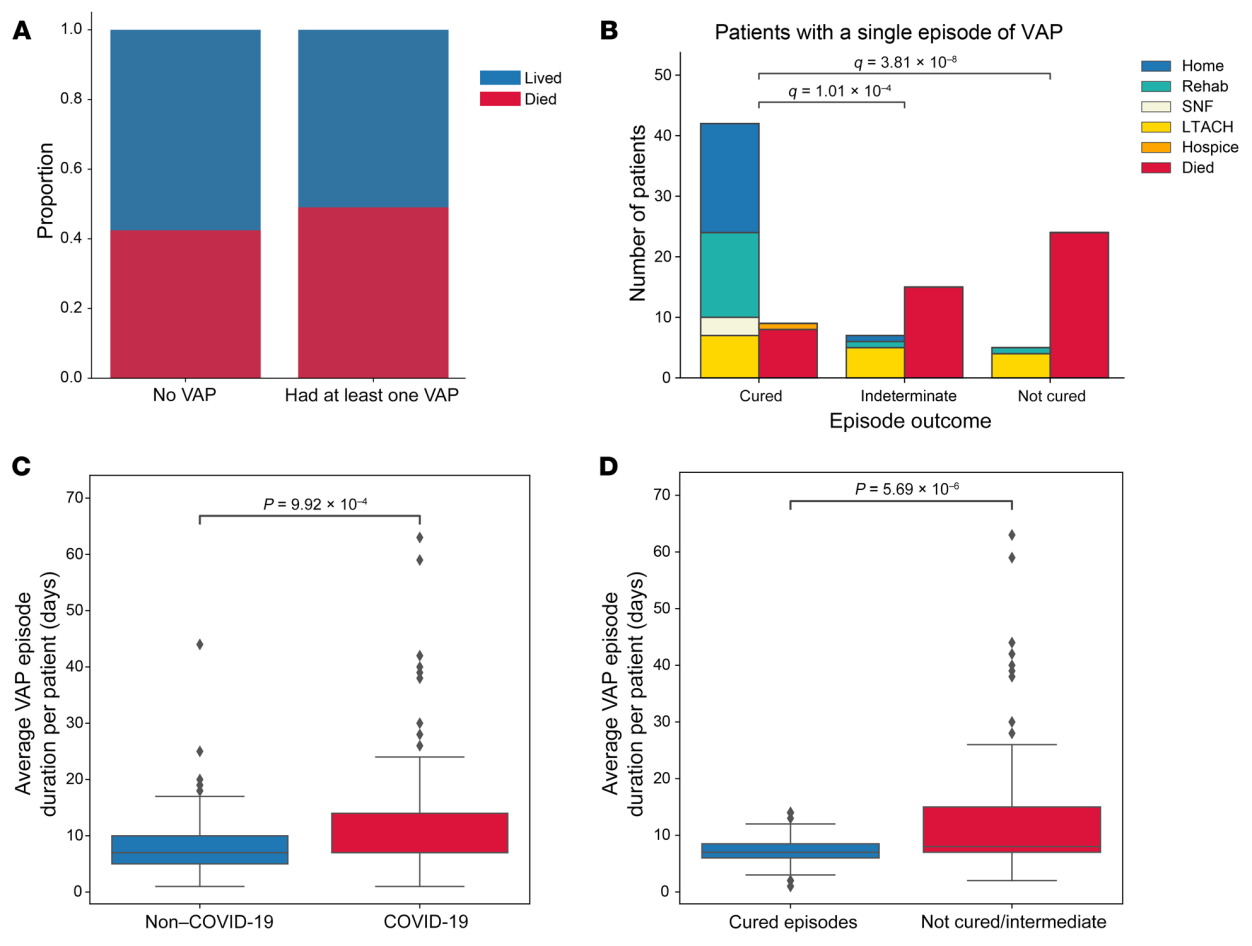
Unresolving VAP is associated with worse outcomes. (**A**) Mortality associated with at least 1 episode of VAP. (**B**) Outcomes for patients who experienced 1 episode of VAP that was cured, of indeterminate cure status, or that was not cured by day 14 following diagnosis. Outcomes are displayed in 2 columns: the first column aggregates favorable discharge dispositions (home, rehabilitation, SNF, LTACH); the second column aggregates unfavorable discharge dispositions (hospice, died). (**C**) VAP episode duration for patients with COVID-19 compared with patients without COVID-19. (**D**) VAP episode duration for patients who were cured or not cured or of indeterminate cure status. For the box-and-whisker plots, the box shows quartiles and the median, and whiskers show minimum and maximum values except for outliers, which are shown as individual data points. Numerical values were compared using the Mann-Whitney *U* test with FDR correction using the Benjamini-Hochberg procedure. Categorical values were compared using Fisher’s exact test with FDR correction using the Benjamini-Hochberg procedure. A *q* value of less than 0.05 was the threshold for statistical significance.

Overall, mortality was not significantly different in patients who developed VAP compared with those who did not (Figure 8A). To further explore the association between VAP and ICU outcomes, we used the validated clinical adjudication results from the SCRIPT study to compare patients with successful treatment of VAP (cured) with those who experienced an indeterminate outcome or unsuccessful treatment (not cured). Examining these endpoints among patients who had only a single VAP episode, we found that mortality was lowest among patients with successful treatment (cured), intermediate among those with an indeterminate outcome, and highest among those with unsuccessful treatment (not cured) (Figure 8B). Among these patients, the rate of unfavorable outcomes (hospice or death) was 17.6% in patients with a cured episode and 76.5% in patients with unsuccessful treatment (intermediate or not cured episode, *P* < 0.001). We also observed a similar pattern among the subset of patients with COVID-19 (Supplemental Figure 16A). Patients with COVID-19 experienced longer durations of VAP episodes (Figure 8C). Unresolving VAP episodes (patients with an indeterminate outcome or who were not cured) were of longer duration than cured episodes (Figure 8D). Since survival is included in our definition of successful VAP treatment, we performed a sensitivity analysis on VAP episodes experienced by patients who survived for at least 14 days after their VAP diagnosis. Even in this group, biased toward better outcomes, we found that unresolving VAP was associated with a higher mortality rate (Supplemental Figure 16B).

### CarpeDiem corroborates the clinical adjudication analysis, identifying an association between unresolving VAP episodes and transitions to unfavorable clinical states associated with a higher hospital mortality rate.

**Figure 9:**
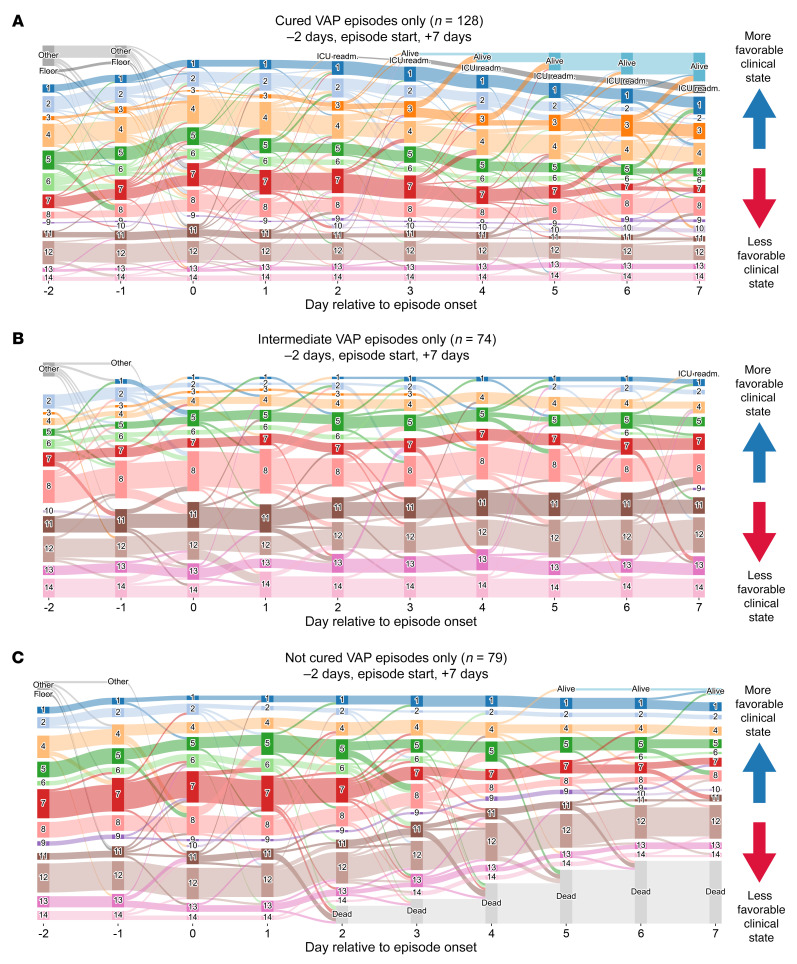
Trajectory analysis reveals that unresolving VAP is associated with transitions to progressively unfavorable clinical states. On these Sankey diagrams, day 0 represents the day that a BAL procedure was performed to evaluate VAP adjudicated as (**A**) cured, (**B**) indeterminate, or (**C**) not cured. More favorable (lower mortality) clinical states are at the top of the graphs, with leaving the ICU alive being the highest, and less favorable (higher mortality) clinical states are at the bottom, with death being the lowest. Graphs start at 2 days prior to the onset of the episode; patients who were not in our ICU are labeled as “Other” (patients who were received in external transfer or chronically ventilated patients) or “Floor” (within 48 hours of extubation or chronically ventilated patients). readm., readmission.

**Figure 10:**
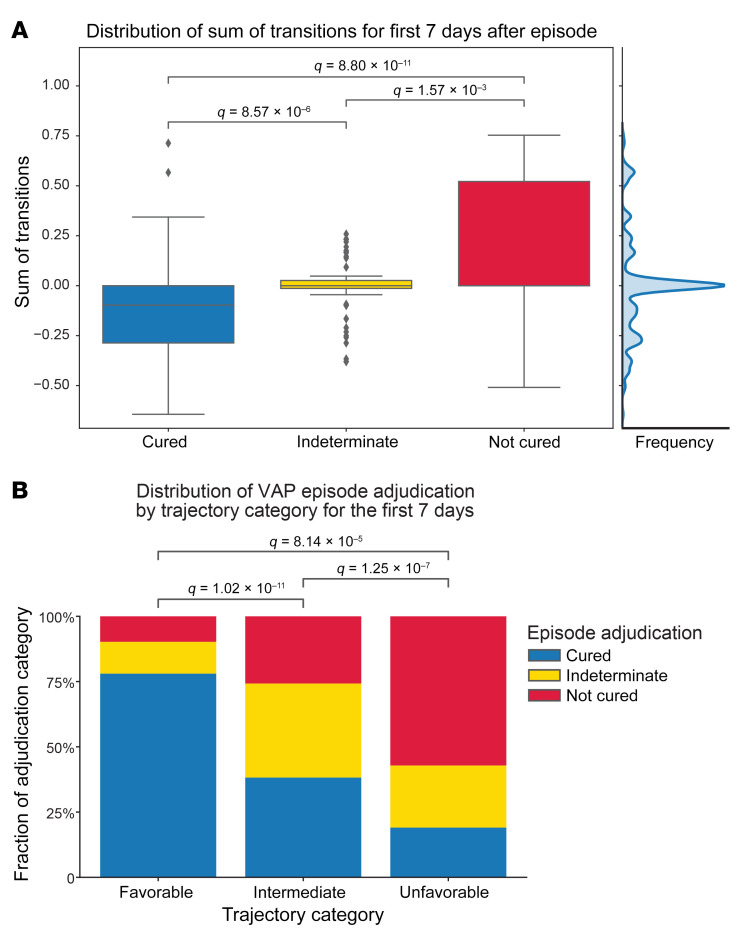
Unresolving VAP episodes are associated with unfavorable clinical states. (**A**) Distribution of the sum of transitions for the 7 days following VAP diagnosis by episode outcome, identifying a breakpoint of 0.1 in the middle of the distribution (shown by the cumulative data histogram along the right axis). Higher sums of transitions reflect transitions to unfavorable (higher mortality) clusters. (**B**) Proportion of VAP episode outcomes in each trajectory category. Trajectories were grouped into favorable (sum of transitions < –0.1), indeterminate (–0.1–0.1), and unfavorable (>0.1) categories. For box-and-whisker plots, the box shows quartiles and the median, and whiskers show minimum and maximum values except for outliers, which are shown as individual data points. Numerical values were compared using the Mann-Whitney *U* test with FDR correction using the Benjamini-Hochberg procedure. Categorical values were compared using χ^2^ tests with FDR correction with the Benjamini-Hochberg procedure. A *q* value of less than 0.05 was the threshold for statistical significance.

We then used the transition analyses provided by *CarpeDiem* to test whether unresolving VAP was associated with a subsequent trajectory toward progressively unfavorable clinical states. To visualize the transitions surrounding the diagnosis of VAP, we generated Sankey diagrams that show the clinical state and transitions encountered before and after the diagnosis of VAP. Successful treatment of VAP was associated with a higher likelihood of favorable subsequent transitions (Figure 9A). In contrast, indeterminate episodes demonstrated a flat trajectory (Figure 9B). Not-cured episodes were associated with a greater risk of unfavorable subsequent transitions (Figure 9C and Supplemental Figure 17). The robustness of the propensity for patients with cured VAP to undergo more favorable transitions than patients without cured VAP was confirmed in subsampling analysis (Supplemental Figure 13E). We then used the sum of transitions occurring in the 7 days following a diagnosis of VAP as a summative measure of trajectory and examined the distribution of trajectories to define favorable, intermediate, and unfavorable trajectory categories (Figure 10A). Favorable trajectories were significantly enriched in cured VAP episodes with significantly higher proportions of indeterminate and not-cured episodes in intermediate and unfavorable trajectory categories, respectively (Figure 10B). Finally, we examined the trajectory categories preceding a VAP diagnosis compared with the average inter-day trajectory across the cohort. We identified an increase in unfavorable transitions 1 day ahead of a VAP diagnosis, presumably reflecting the clinical events that prompted the diagnostic BAL procedure, that was not associated with the duration of the ensuing VAP episode (Supplemental Figure 18, A and B).

To assess whether the same associations could be revealed to be revealed independently of the *CarpeDiem* approach, we added flags denoting the development of VAP and its outcome to a standard model of ICU mortality prediction based on clinical parameters measured early (in the first 2 days) of ICU admission. Using gradient boosting, we found only a nominal increase in the predictive ability of early clinical parameters with addition of the VAP flags (Supplemental Figure 19A). These findings are possibly explained by the disconnect between the clinical parameters measured early in a clinical course and the fact that VAP, by definition, occurs later in an ICU stay. Expectedly, the same clinical parameters applied to the median 2 days or final 2 days of the ICU stay had intermediate and excellent predictive capability, respectively, but were similarly unmodified by the addition of the VAP flags (Supplemental Figure 19, B and C).

## Discussion

The ICU course of patients with severe SARS-CoV-2 pneumonia is more than twice as long as the duration among similarly ill patients with pneumonia and respiratory failure due to other etiologies (1–15). Despite significantly longer durations of critical illness, the mortality rate for patients with COVID-19 is similar to that of patients with other causes of pneumonia and respiratory failure (2–4, 6). We and others have reported unexpectedly high rates of VAP complicating the ICU course of patients with SARS-CoV-2 pneumonia (7, 23). In this large prospective, observational cohort study, we used state-of-the-art microbiological analysis of serially-collected BAL samples (7, 30, 37) over the course of the ICU stay combined with validated clinical adjudications to identify VAP episodes and clinical endpoints. We found that unresolving episodes of VAP were associated with mortality, including among patients with COVID-19. Accordingly, we suggest that the discordance between ICU LOS and mortality for patients with severe SARS-CoV-2 pneumonia resulted from a low mortality rate attributable to the primary viral pneumonia that was offset by an increased risk of mortality from unresolving VAP or other ICU complications.

Our cohort included large numbers of patients with COVID-19 and similarly ill patients with pneumonia secondary to other pathogens, providing an opportunity to determine whether and how VAP differentially contributes to outcomes in patients with COVID-19. Compared with patients with pneumonia secondary to other pathogens, we found that patients with COVID-19 had a longer duration of mechanical ventilation and ICU stay and higher rates of VAP, yet a similar mortality rate. Because VAP, the duration of mechanical ventilation, and mortality are interrelated, we developed a data-driven, machine-learning approach to disentangle these features. *CarpeDiem* uses data extracted from the EHR to discretize days in the ICU and generate clusters of patient-days with similar physiological and laboratory features, paralleling the practice of daily ICU rounds. As key clinical data drove the *CarpeDiem* algorithm, the resulting clusters represented clinical states that were associated with differential hospital mortality. As patients improve or worsen, they undergo transitions to more favorable (lower mortality) or less favorable (higher mortality) clinical states. We reasoned that if multiple organ failure drives prolonged ICU LOS among patients with COVID-19, *CarpeDiem* would identify frequent transitions between clinical states associated with more organ failures. Instead, *CarpeDiem* showed that the long ICU LOS among patients with SARS-CoV-2 pneumonia was attributable to significantly longer stays in clinical states primarily characterized by severe hypoxemic respiratory failure. When normalized for ICU LOS, patients with SARS-CoV-2 pneumonia experienced fewer transitions between clinical states than did other patients over the course of their ICU stay. This finding provides clinical support for emerging models of SARS-CoV-2 pneumonia pathobiology. In these models, severe SARS-CoV-2 pneumonia results from a slowly progressive but spatially localized pulmonary infection that unfolds over days to weeks (6, 16, 38), leading to prolonged respiratory failure and higher rates of VAP.

Guidelines adopted by professional societies recommend a host of interventions to prevent and treat known or suspected VAP in patients requiring mechanical ventilation, implicitly acknowledging the importance of VAP in determining outcomes (39, 40). Nevertheless, we are unaware of prior studies demonstrating an association between unresolving VAP with poor ICU outcomes. We used a rigorous clinical and microbiological adjudication procedure to show that unresolving VAP was associated with mortality, including in patients with SARS-CoV-2 pneumonia. Furthermore, *CarpeDiem* demonstrated that unresolving VAP was associated with transitions toward unfavorable clinical states, providing independent, complementary, and unsupervised support for our adjudication procedures and findings. Perhaps as importantly, we found that successfully treated VAP was associated with improved outcomes and favorable transitions in all patients with severe respiratory failure. These findings suggest that improved strategies to diagnose and successfully treat VAP episodes, including pathogen-directed therapy guided by BAL fluid analysis, may improve ICU outcomes.

The importance of VAP as a driver of mortality in patients with COVID-19 has been underestimated, probably because bronchoscopic sampling has been uncommon during the pandemic, the use of antibiotics is ubiquitous, and clinical criteria and biomarkers do not accurately distinguish between primary SARS-CoV-2 pneumonia and secondary bacterial pneumonia (41). For example, only 1 episode of secondary pneumonia was reported in the 403 patients included in the Randomized Embedded Multifactorial Adaptive Platform for Community-Acquired Pneumonia (REMAP-CAP) trial of hydrocortisone for COVID-19, and no episodes were reported in the 6,425 patients included in the RECOVERY trial of dexamethasone therapy for COVID-19 (1, 15). If unresolving episodes of VAP, rather than the primary viral pneumonia, contribute to mortality in a substantial fraction of patients with severe SARS-CoV-2 pneumonia, it might explain why therapies that attenuate the host response (e.g., corticosteroids, IL-6 receptor antagonists, JAK2 inhibitors, and calcium release–activated calcium [CRAC] channel inhibitors) are more effective when administered early in the clinical course, before patients are intubated and at risk for VAP (1, 34, 42–44).

Our study has important limitations. First, as ours is an observational study, we cannot exclude unmeasured confounders that link unresolving VAP to poor outcomes. Other processes of care, such as ventilator and antibiotic management strategies, and host factors, such as exposure to immunomodulatory therapies and alterations in the microbiome, probably drive VAP outcomes. Second, we used state-of-the art clinical microbiological analysis of distal lung samples to diagnose VAP, and we have shown that clinicians in our center use this information to optimize, narrow, or discontinue antibiotic therapy (7), minimizing its harmful effects (45). The reasons underlying the failure of appropriate antimicrobial therapy in some patients cannot easily be determined from our study, raising important questions about the drivers of unresolving VAP despite targeted antimicrobial therapy. Potential drivers of unresolving VAP include pharmacodynamic and pharmacokinetic properties of pathogen-targeted antibiotics, dysregulated microbiome composition, and an inappropriate balance between host immune responses that favor ongoing inflammation and injury versus resolution and repair. Further studies of the pathogen, microbiome, and host response using high-resolution, next-generation sequencing approaches applied to BAL fluid and spatial profiling applied to lung tissue may provide insights into these mechanisms. Ultimately, causal validation of these mechanisms will need to come from in vitro systems, experimental animal models of pneumonia, and randomized, controlled trials in patients. Third, it is important to note that the clustering tools used in *CarpeDiem*, necessarily driven by patients with a longer LOS, will generate different clusters as the composition of the cohort changes. Therefore, *CarpeDiem* is primarily useful to compare ICU diagnoses, interventions, and complications within a single cohort, while application of the clinical states from 1 cohort to another, or prospective assignment of clinical states to new data in the same cohort, remains to be investigated. In our analysis of patients enrolled in a randomized, controlled trial of sarilumab, who were also included in our cohort, we demonstrated the potential utility of the *CarpeDiem* approach for generating hypotheses about mechanisms that may underlie negative findings in the trial. In our example, the results suggested that the negative outcome might have resulted from a lack of repeated sarilumab dosing. If confirmed in a larger numbers of patients, these results might inform the design of subsequent trials. Importantly, *CarpeDiem* uses only data collected as a part of routine clinical care; therefore, it could be retrospectively applied to EHR data from multiple centers to analyze the results of this or other clinical trials in the ICU. Fourth, *CarpeDiem* uses a limited number of parameters to define clinical states, potentially neglecting important determinants of outcome and information that might be found in missing data (e.g., reduced monitoring and ordering of laboratory tests as patients improve or move toward comfort-focused care). Similarly, intermittently measured biomarkers associated with outcomes, for example those used by Calfee et al. to define hyper- and hypoinflammatory states in the ICU, are incompletely represented in *CarpeDiem* (46). Future iterations of the tool can incorporate these data with a goal of improving the association between clinical states and mortality in both observational and interventional studies. To this end, we have made deidentified data from the SCRIPT data set, as well as detailed code, freely available to the research community.

## Methods

### Study setting.

Patients were enrolled in the SCRIPT Systems Biology Center, a single-site, prospective cohort study of patients hospitalized in the ICUs of Northwestern Memorial Hospital (NMH) with suspected severe pneumonia (severe pneumonia defined as lower respiratory tract infection requiring mechanical ventilation), all of whom underwent at least 1 BAL procedure. A subset of patients were co-enrolled in a randomized, placebo-controlled trial of the IL-6 receptor antagonist sarilumab (NCT04315298).

### Study procedures.

ICU physicians at NMH routinely obtain bronchoscopic or nonbronchoscopic BAL samples from mechanically ventilated patients whenever pneumonia is suspected (47). In SCRIPT, the patients were screened for enrollment when the clinical team decided to perform the first BAL procedure. For all BAL samples, NMH clinical laboratories perform quantitative bacterial culturing and antimicrobial susceptibility testing. Many of the samples in SCRIPT were also analyzed by multiplex PCR (BioFire FilmArray Pneumonia [PN] Panel), with results provided to the clinical team within 3 hours. We previously reported that our physicians initiate guideline-recommended antimicrobial therapy when pneumonia is suspected and use data obtained from analysis of BAL fluid to appropriately narrow or discontinue empirical guideline-recommended antimicrobial therapy (7).

### Data extraction and analysis.

Demographics, clinical data, and outcome data were extracted from the EHR via the Northwestern Medicine Enterprise Data Warehouse (48). For the *CarpeDiem* machine-learning approach, we trialed 3 different computational strategies that involved hierarchical clustering of 44 clinical features. We chose the number of clusters by optimizing clinical interpretability and reasonable between-clusters differential mortality. UMAP (49) was used for visualization. We externally validated the *CarpeDiem* approach in a suspected pneumonia cohort derived from the MIMIC-IV database (33), using code from the MIMIC Code Repository (50). Selection criteria in the MIMIC-IV cohort included admission to and discharge from a medical ICU, respiratory failure requiring mechanical ventilation, and pneumonia as defined by International Classification of Diseases, Ninth Revision (ICD-9) codes. We used XGBoost (51) to model outcomes based on clinical features from the first 2 days (similar to most clinical prediction models), the median 2 days, and the last 2 days of the admission. See Supplemental Methods for further details.

### Definition of pneumonia episodes.

A panel of 6 critical care physicians used a prospectively generated, standardized score sheet (Supplemental Data 2) to manually review each patient’s EHR, including clinical notes, to identify and categorize pneumonia episodes and adjudicate whether these episodes were successfully treated (i.e., resolved). A detailed description of our adjudication protocol is available (52). Pneumonia episodes were captured up to 99 days following the enrollment BAL procedure and categorized as nonpneumonia controls, other pneumonia (bacterial), other viral pneumonia, or COVID-19. By definition, VAP was considered to be an incident pneumonia that was diagnosed by a BAL performed after at least 48 hours of mechanical ventilation (39); only VAP episodes that were adjudicated to be due to bacteria were included in the analysis. VAP duration was defined as the time interval between the diagnostic BAL procedure and clinical cure, discontinuation of antibiotics, or death, whichever was the shortest. Endpoints for VAP episodes were adjudicated on days 7/8, 10, and 14 following the diagnostic BAL procedure. See Supplemental Methods for detailed definitions.

### Statistics.

Numerical values were compared using Mann-Whitney *U* tests with FDR correction using the Benjamini-Hochberg procedure. Categorical values were compared using Fisher’s exact tests with FDR correction using the Benjamini-Hochberg procedure. A *P* value or *q* value of less than 0.05 was the threshold for statistical significance.

### Study approval.

This study was approved by the Northwestern University IRB (study ID STU00204868). The sarilumab trial was approved by the Northwestern University IRB (study ID STU00212239).

### Code and data availability.

Programming was performed in Python (version 3.9). A detailed description of all data extraction and computational procedures, including code, are available at https://github.com/NUSCRIPT/carpediem and in Supplemental Methods. A deidentified version of all SCRIPT cohort data used in this manuscript is available on PhysioNet at https://doi.org/10.13026/5phr-4r89 (53, 54). A demo interactive data browser illustrating the features of *CarpeDiem* is available on our website (https://nupulmonary.org/carpediem), and the full browser is available on PhysioNet.

## Author contributions

CAG, NSM, TS, GRSB, RGW, AVM, and BDS conceived, designed, interpreted results, and wrote/edited the manuscript. CAG, NSM, and TS performed programming and analysis. CAG, CP, JMW, JMK, RGW, and BDS performed clinical pneumonia episode adjudication. HKD and AD enrolled patients and compiled clinical adjudication data. AP, PN, RAG, CAG, MK, LR, DS, JS, and YL compiled the data set. CAG, NSM, TS, MK, LR, DS, and JS directly accessed and verified the underlying data reported in the manuscript. All authors confirm that they had full access to all the data in the study and accept responsibility for the submission for publication. All authors read and approved the final draft of the manuscript. The order of the 3 co–first authors was determined on the basis of their contributions to the writing of the manuscript.

## Supplementary Material

Supplemental data

Supplemental data 2

ICMJE disclosure forms

Supplemental table 3

## Version 1 04/27/2023

In-Press Preview

## Version 2 06/15/2023

Electronic publication
