# Supplementary material for: Machine learning links unresolving secondary pneumonia to mortality in patients with severe pneumonia, including COVID-19
Source: J Clin Invest. 2023 Jun 15;133(12):e170682. doi: 10.1172/JCI170682 (PMC10266785; doi:10.1172/JCI170682)
Supplement: Supplemental data [file jci-133-170682-s091.pdf]

Supplemental Materials

Machine learning links unresolving secondary pneumonia to mortality in patients with severe pneumonia, including COVID-19

Catherine A. Gao[1] *, Nikolay S. Markov[1] *, Thomas Stoeger[2]*, Anna Pawlowski[3], Mengjia Kang[1], Prasanth Nannapaneni[3], Rogan A. Grant[1], Chiagozie Pickens[1] , James M. Walter[1] , Jacqueline M. Kruser[1,4], Luke Rasmussen[5], Daniel Schneider[3], Justin Starren[5], Helen K. Donnelly[1], Alvaro Donayre[1], Yuan Luo[5], GR Scott Budinger[1,6] **, Richard G. Wunderink[1,6] **, Alexander V. Misharin[1,6] **, Benjamin D. Singer[1,6] **,
The NU SCRIPT Study Investigators

*co-first authors
**co-senior authors

[1] Division of Pulmonary and Critical Care Medicine, Department of Medicine, Northwestern University Feinberg School of Medicine, Chicago, IL, USA
[2] Department of Chemical and Biological Engineering, Northwestern University, McCormick School of Engineering, Evanston, IL, USA
[3] Northwestern Medicine Enterprise Data Warehouse, Northwestern University Feinberg School of Medicine, Chicago, IL, USA
[4] Division of Allergy, Pulmonary and Critical Care, Department of Medicine, University of Wisconsin School of Medicine and Public Health, Madison, WI, USA
[5] Division of Health and Biomedical Informatics, Department of Preventive Medicine, Northwestern University Feinberg School of Medicine, Chicago, IL, USA
[6] Simpson Querrey Lung Institute for Translational Science (SQLIFTS), Northwestern University Feinberg School of Medicine, Chicago, IL, USA

**Corresponding authors:
GR Scott Budinger, Richard G. Wunderink, Alexander V. Misharin, Benjamin D. Singer
303 E. Superior Street
Simpson Querrey, 5th Floor
Chicago, Illinois 60611, USA
Emails: s-buding@northwestern.edu, r-wunderink@northwestern.edu, a-misharin@northwestern.edu, benjamin-singer@northwestern.edu

Supplemental Methods

Cohort and patient information. Our cohort included mechanically-ventilated patients admitted to an intensive care unit (ICU) who were enrolled in the Successful Clinical Response in Pneumonia Therapy (SCRIPT) study from June 2018 through March 2022 at Northwestern Memorial Hospital (NMH). All patients in the cohort, including those with COVID-19, were cared for in dedicated ICUs by clinical teams led by physicians credentialed in intensive care medicine. We analyzed only patient stays with a hospital discharge disposition and for which all pneumonia episodes had full clinical adjudication. We assigned patients to one of four groups (non-pneumonia control, other pneumonia [bacterial], other viral pneumonia, or COVID-19) based on their initial SCRIPT enrollment bronchoalveolar lavage (BAL) results, as established by BAL fluid analysis and assessed by ICU physician adjudicators (see below). Ages greater than 89 were grouped together per Safe Harbor guidelines (1) and reported as age 91, as has been done in other datasets (2). Racial groups with fewer than five individuals were classified as 'Unknown or Not Reported' to protect patient anonymity. Patients who underwent lung transplantation for persistent respiratory failure were categorized as having died. Comorbidities were compiled using ICD codes present on hospital admission, mapped to Charlson Comorbidity Index. In binarized outcomes, discharge to hospice or death were categorized as an outcome of 'Died,' whereas discharge to Home, Rehab, Skilled Nursing Facility, or Long-term Acute Care Hospital were categorized as 'Lived.' Supplemental Tables were generated using *tableone* (3).

Study procedures. Clinicians at NMH use BAL fluid cell count and differential (4, 5) and serum levels of procalcitonin (6) to assess the pretest probability of pneumonia secondary to bacterial pathogens while awaiting final quantitative culture results. BAL fluid amylase is measured to estimate the risk of aspiration (7). Physicians are encouraged to order testing of urine for *S. pneumoniae* and *L. pneumophila* antigens, testing of BAL fluid for acid-fast bacilli and fungal culture, and testing of BAL fluid or serum for aspergillus galactomannan and 1,3 β-D glucan levels when clinically appropriate.

ICU stay information. Electronic health record (EHR) data are compiled by the Northwestern Medicine Enterprise Data Warehouse (EDW) (8), the primary data repository for clinical data at Northwestern Medicine.

Approximately 150 data sources (including the main EHR system, Epic) are loaded into the EDW on a nightly basis. The data are primarily loaded using Microsoft technologies (Visual Studio, SSIS, etc.) and scheduled to load via the SQL Server Job Agent. Data engineers and architects on the EDW team then combine the data sources using custom SQL scripts, Visual Studio, SSIS, etc. to create datamarts. Analysts on the EDW team then work with the datamarts to create reports, dashboards, and extracts validated with clinician input. Patients who had multiple ICU stays during the same hospitalization had their stays numbered consecutively but did not contribute data between their ICU stays. Multiple hospitalizations of the same patient are reported as separate patients, as SCRIPT enrollments are unique to each hospitalization. The dates of each per-day datapoint were removed and replaced with a day relative to the beginning of the ICU stay (initial day is day 1, with next day starting at 12:00 a.m.) for each ICU admission occurring during a hospitalization such that no dates are in the final dataset per Safe Harbor guidelines (1).

Clinical parameters. First, we manually selected 44 clinical features that we considered representative of those that physicians would consider during daily ICU rounds, including the components of the Sequential Organ Failure Assessment (SOFA) score. We compiled the status of intubation, extracorporeal membrane oxygenation (ECMO), acute renal replacement therapy (hemodialysis [HD], and continuous renal replacement therapy [CRRT]), sedation parameters (Glasgow Coma Scale [GCS] subscores of eye opening, motor response, verbal response, and Richmond Agitation Sedation Scale [RASS]), lung injury (PaO_2/FiO_2 ratio, PaO_2, arterial pH, $PaCO_2$, PEEP, FiO_2, plateau pressure, lung compliance, and oxygen saturation), hemodynamics (norepinephrine rate in mcg/kg/min, a flag for norepinephrine, mean arterial pressure, systolic and diastolic blood pressure, lactic acid, hemoglobin, and bicarbonate), renal (creatinine, urine output, and aforementioned HD and CRRT flags), inflammatory markers (WBC count, neutrophil count, platelets, procalcitonin, C-reactive protein, D-dimer, lactate dehydrogenase, ferritin, bilirubin, albumin, and lymphocytes), vital signs (temperature, heart rate, respiratory rate), and ventilator instability (number of ventilator respiratory rate changes, positive end-expiratory pressure (PEEP) changes, and FiO_2 changes per day). When multiple measurements were available for the same day, they were aggregated to produce a single value for a given day (worst value for SOFA parameters and mean for others). Outliers were removed prior to aggregation by using predefined ranges for each

measurement. When no measurements were available, we reported it as not available (NA). For patients on ECMO, we set PaO_2/FiO_2 ratio to NA; for patients on HD or CRRT, we set creatinine to NA, as these parameters are unreliable when patients are receiving these interventions. Full details are available in our code at https://github.com/NUSCRIPT/carpediem.

Data analysis. We used the following unsupervised strategy, referred to as "Similarity." First, as above, we manually selected 44 clinical features that we considered representative of those that physicians would consider during daily ICU rounds, including the components of the SOFA score. Second, we masked some values that we considered out of range. Third, we summarized all values per patient per day, using average or worst value, depending on the feature, and marking absent values as NA. Fourth, we performed pairwise Pearson correlations among all per-day features and considered features with a Pearson correlation above 0.7 to be highly related. Fifth, we normalized each feature with percentile normalization and set tied values to the average percentiles. Sixth, we combined highly related features to a single feature by taking the mean percentile across the related features. Seventh, we created a similarity matrix between all pairs of patient-days by computing the Pearson correlation among features that had values present for both patient-days. Finally, we computed Euclidean distances on the similarity matrix and performed hierarchical clustering on individual patient-days using Ward's approach (9). Additionally, we considered two other strategies: "Ranked-Euclidean" and "Normalized-Euclidean." During the sixth step, "Ranked-Euclidean" reweights highly related features by dividing their values by the square root of the number of related features, then skips the seventh step, and during the eighth step applies an NA-robust implementation of Euclidean distances as described by Dixon (10), available in the *scikit-learn* package (11). "Normalized-Euclidean" follows the procedure of "Ranked-Euclidean," but at the fifth step replaces a general percentile normalization by winsorizing, followed by $\log_2$ or percentile normalization, depending on specific data input. For each step, see code and ensuing sections for full details.

Correlation weighting. Some measurements in our dataset displayed a high correlation due to mathematical or physiological coupling (e.g., plateau pressure, PEEP and lung compliance; PaCO2 and bicarbonate). To make such correlated measurements contribute comparable value to the difference between data points as an

independent measurement, we applied correlation weighting (**Supplemental Figure 3B**). We defined related measurement groups as measurements that have Pearson correlation greater than 0.7 with at least one other measurement. We combined related features to a single feature by taking the mean percentile across the related features and then considering the percentile across these new values.

Distance function. We created a similarity matrix between all pairs of patient-days by computing the Pearson correlation among features that had values present for both patient-days after correlation weighting (**Supplemental Figure 3C**). We computed Euclidean distances on the similarity matrix.

Clustering and the number of clusters. We were interested in describing the highest number of clinical states that were clinically interpretable and produced reasonable between-cluster separation in mortality rates. After computing the distance metric between all pairs of data points, we performed hierarchical clustering using Ward's method (12) to generate a dendrogram. To estimate what number of clusters best describes our data, we assessed how data points were grouped into 3 up to 40 clusters using a custom metric for differential mortality and by visually inspecting progressive splits of the dendrogram on a heatmap. We posited that clinically relevant clustering of patient-days would reveal clinical states that are associated with differential mortality. Thus, our custom metric was generated by computing the fraction of all pairs of clusters that had statistically significant differences in mortality using Fisher's exact test with $p < 0.01$. Cluster mortality was computed as the number of patients with a 'Died' binarized outcome among all patients who had at least one patient-day assigned to a cluster. We also examined the cluster output using silhouette scores (13). We found high silhouette scores when we only used a few features with YES/NO flags, but these clusters did not perform well when separating the cohorts by mortality. Thus, there appears to be a tradeoff between cohesion and the ability to generate granular information relating sparse ICU events like VAP and trajectories between mortality-associated clusters (**Supplemental Figure 5C-D**).

Alternative clustering strategies. We called the above data processing and clustering strategy "Similarity." We also tested two additional strategies, which we called "Ranked-Euclidean" and "Normalized-Euclidean." The

“Ranked-Euclidean” strategy differs from “Similarity” by performing correlation weighting differently and by using a different distance function. “Normalized-Euclidean” strategy had alternative feature normalization, correlation weighting, and distance function procedures. We used the same methods as described in *Clustering and the number of clusters* to find the best clustering strategy, and also to choose the optimal threshold for highly correlated features.

“Ranked-Euclidean” alternative strategy. Highly correlated features were reweighted by dividing feature values by square root of the features’ group sizes. We computed distances between patient-days as Euclidean distance directly on the normalized and reweighted features, without computing a similarity matrix. We used an NA-robust implementation of Euclidean distances as described by Dixon (10), available in *scikit-learn* (11).

“Normalized-Euclidean” alternative strategy. For flag and score features, we performed percentile normalization as above. For other features, we applied additional steps. We applied winsorizing with a limit of 0.01, either one-sided or two-sided, depending on the measurement boundaries (e.g., oxygen saturation has a natural maximum of 100%, so we adjusted only the lowest 1% of values). For features that displayed exponential distribution, we performed $\log_2$ transformation. We then linearly normalized values to a 0–1 range. We reweighted correlated features and computed distances between all patient-days as in the “Ranked-Euclidean” strategy.

Robustness against exclusion of individual patient hospitalizations. To evaluate the robustness of our clustering to minor perturbations, we randomly removed 100 different hospitalizations and performed the “Similarity” strategy. Across these randomizations, we found that individual patient-days would fall into clusters with similar associated mortality (**Supplemental Figure 5B**).

Robustness of main conclusions against exclusion of multiple patients. To evaluate the robustness of biological conclusions, we randomly selected 80% of all patients prior computing distances between patient days. The specific conclusions tested this way were: the number of normalized transitions among patients with and without COVID-19 (**Supplemental Figure 13A-B**), enrichment in patients with COVID-19 in clusters having a higher

respiratory score (**Supplemental Figure 13C-D**), and our findings on unresolved VAP having a larger number on unfavorable transitions than resolved VAP (**Supplemental Figure 13E**).

Applicability of distances as metric for clustering. NA-robust implementation of Euclidean distances used for the “Ranked-Euclidean” and “Normalized-Euclidean” does not guarantee preservation of the triangle inequality. To test the behavior of these strategies, we randomly sampled 10 million triplets of patient-days. Of these 10 million, we observed 0, 245, and 456 triplets that violated the triangle inequality when following the “Similarity,” “Ranked-Euclidean,” and “Normalized-Euclidean” strategies, respectively.

Visualization. To visualize clusters as distinct and recognizable clinical states, we ordered the clusters by increasing mortality, summarized clinical measurements for each cluster for each group of clinical measurements (neurologic, respiratory, shock, renal, inflammatory, and ventilator instability) and plotted the result as a heatmap (**Figure 2B**). Each measurement contributed to its group either directly (higher-is-worse) or inversely (lower-is-worse) so that all groups have higher-is-worse semantics. The four flags (ECMO, intubation, CRRT, and HD) were weighted with their original 0/1 score, whereas other parameters were weighted with their post-normalization value. Features were normalized using min-max normalization and plotted using spider plots for the six grouped features.

To visualize patient-days as clusters, we used the UMAP algorithm (14) to obtain 2-dimensional representation of our 44-dimensional dataset (**Supplemental Figure 6**). To overcome UMAP’s inability to handle NAs, we disabled the *check_array* function, computed the kNN graph based on our predefined distance matrix (see *Distance function* above), and obtained UMAP 2D embedding by using the precomputed kNN graph. For final visualization we used seven nearest neighbors and UMAP hyperparameters min_dist=0.2.

All plots were created in Python with matplotlib version 3.4.3 and seaborn version 0.11.2 libraries. Full details are available in our code at https://github.com/NUSCRIPT/carpediem.

Data browser and example trajectory generation. Trajectories formed by transitions between *CarpeDiem*-defined clinical states are a valuable resource for clinicians. To facilitate data exploration, reflection, and insight, we developed an interactive visualization for our dataset. Currently, the interface allows for patient-based data exploration; the user can select a patient and view the clinical states associated with each day of the patient's ICU stays, together with the clinical measurements. BAL sample and pneumonia episode information (see below) is overlaid onto the patient trajectory timeline. We used d3.js version 7.1.1 to create the interactive visualization. One of the patient trajectories is plotted in **Supplemental Figure 6J** with relevant clinical events manually annotated. A demonstration browser can be viewed at https://nupulmonary.org/carpediem/, and a full browser is available on PhysioNet at https://doi.org/10.13026/5phr-4r89.

Modeling. We used XGBoost (15) to model outcomes based on clinical features taking the worst values from the first two days (consistent with commonly used ICU prediction scores), the median two days, and the final two days in the patient's stay. To this baseline model, we added a flag noting whether patients developed VAP during hospitalization and a flag noting whether this VAP's outcome was indeterminate or not cured (as opposed to cured). Confidence intervals were generated via bootstrapping. Models were trained using an 80/20 train/test split, and parameters were tuned to optimize test run performance.

Description of clinical adjudication process. A detailed description and validation of the clinical adjudication process is available (16). Each case was reviewed independently by two physicians with discrepant adjudications settled by a third. If the third review remained discrepant, the episode was discussed at a meeting of the panelists to determine a consensus decision. Outcomes for bacterial VAP episodes were adjudicated at day 7-8, day 10, and day 14 following the diagnostic BAL procedure beginning with the episode that prompted enrollment in SCRIPT. In total, 9,850 patient-days occurred following SCRIPT enrollment. Cure was defined as the ability to survive beyond the duration of antibiotic treatment, the ability to remain off of antibiotics for 48 hours without recurrence or superinfection pneumonia, disappearance of the causative pathogen from BAL fluid or the absence of subsequent samples, absence of bacterial complications (e.g., empyema, lung abscess, endocarditis), and improvement in the clinical manifestations of pneumonia; successful extubation or ventilator liberation was

considered as a cure. Failure was considered if the patient died during the antibiotic treatment course, the development of pneumonia led to a shift to comfort-only care, the patient developed complications such as empyema, lung abscess, or endocarditis or a persistent need for vasopressors or hemodynamic instability until a change in antibiotics. Specific failure modes included persistence, defined as interval recovery of the causative pathogen in a respiratory tract specimen, blood, or pleural fluid or development of an abscess/cavity, empyema, or endocarditis. Failure due to superinfection was defined as recovery of a new pneumonia pathogen while being treated for pneumonia. Indeterminate status indicated persistent inflammation (BAL fluid neutrophilia, fever, elevated white blood cell count without other explanation) or respiratory failure without demonstration of persistence or superinfection as defined above.

References

1. Office for Civil Rights (OCR). Guidance regarding methods for DE-identification of protected health information in accordance with the Health Insurance Portability and Accountability Act (HIPAA) Privacy Rule [Internet]. *Hhs.gov* 2012;https://www.hhs.gov/hipaa/for-professionals/privacy/special-topics/de-identification/index.html. cited September 9, 2022

2. Johnson A et al. MIMIC-IV [Internet]2022; doi:10.13026/7VCR-E114

3. Pollard TJ, Johnson AEW, Raffa JD, Mark RG. tableone: An open source Python package for producing summary statistics for research papers. *JAMIA Open* 2018;1(1):26–31.

4. Walter JM et al. Multidimensional Assessment of the Host Response in Mechanically Ventilated Patients with Suspected Pneumonia. *Am. J. Respir. Crit. Care Med.* 2019;199(10):1225–1237.

5. Grant RA et al. Circuits between infected macrophages and T cells in SARS-CoV-2 pneumonia [Internet]. *Nature* [published online ahead of print: January 11, 2021]; doi:10.1038/s41586-020-03148-w

6. Torres A et al. International ERS/ESICM/ESCMID/ALAT guidelines for the management of hospital-acquired pneumonia and ventilator-associated pneumonia: Guidelines for the management of hospital-acquired pneumonia (HAP)/ventilator-associated pneumonia (VAP) of the European Respiratory Society (ERS), European Society of Intensive Care Medicine (ESICM), European Society of Clinical Microbiology and Infectious Diseases (ESCMID) and Asociación Latinoamericana del Tórax (ALAT) [Internet]. *Eur. Respir. J.* 2017;50(3). doi:10.1183/13993003.00582-2017

7. Weiss CH, Moazed F, DiBardino D, Swaroop M, Wunderink RG. Bronchoalveolar lavage amylase is associated with risk factors for aspiration and predicts bacterial pneumonia. *Crit. Care Med.* 2013;41(3):765–773.

8. Starren JB, Winter AQ, Lloyd-Jones DM. Enabling a Learning Health System through a Unified Enterprise Data Warehouse: The Experience of the Northwestern University Clinical and Translational Sciences (NUCATS) Institute. *Clin. Transl. Sci.* 2015;8(4):269–271.

9. Ward JH. Hierarchical Grouping to Optimize an Objective Function [Internet]. *Paperpile* https://paperpile.com/app/p/9d74dee9-431f-03b6-a0f1-5be2b24b7094. cited September 19, 2022

10. Dixon JK. Pattern Recognition with Partly Missing Data. *IEEE Trans. Syst. Man Cybern.* 1979;9(10):617–621.

11. Pedregosa F et al. Scikit-learn: Machine Learning in Python. *J. Mach. Learn. Res.* 2011;12(85):2825–2830.

12. Ward JH. Hierarchical Grouping to Optimize an Objective Function. *J. Am. Stat. Assoc.* 1963;58(301):236–244.

13. Rousseeuw PJ. Silhouettes: A graphical aid to the interpretation and validation of cluster analysis. *J. Comput. Appl. Math.* 1987;20:53–65.

14. McInnes L, Healy J, Melville J. UMAP: Uniform Manifold Approximation and Projection for Dimension Reduction [Internet]. *arXiv [stat.ML]* 2018;http://arxiv.org/abs/1802.03426. cited

15. Chen T, Guestrin C. XGBoost: A Scalable Tree Boosting System. In: *Proceedings of the 22nd ACM SIGKDD International Conference on Knowledge Discovery and Data Mining*. New York, NY, USA: Association for Computing Machinery; 2016:785–794

16. Pickens CI et al. An adjudication protocol for severe bacterial and viral pneumonia [Internet]. *bioRxiv* 2022; doi:10.1101/2022.10.26.22281461

Supplemental Data File 2. Standardized score sheet used by physician reviewers to adjudicate pneumonia episodes.

Separate file.

Supplemental Table 3. The NU SCRIPT Study Investigators.

Separate file.

Supplemental Table 1. Demographics and outcomes data for the cohort, grouped by pneumonia category.
[A] Racial groups with fewer than five individuals were classified as 'Unknown or Not Reported' to protect patient anonymity.
[B] One patient did not have BMI documented.
[C] Total days intubated and total ICU days include only days at our hospital and do not capture intubation duration or ICU LOS at a transferring hospital.
[D] Died included those who died or underwent lung transplantation for refractory respiratory failure.
BMI = body mass index, APS = Acute Physiology Score (score calculated from worst value within the first two ICU days), SOFA = Sequential Organ Failure Assessment (score calculated from worst value within the first two ICU days).

Feature	Overall	Non-Pneumonia Control	Other Pneumonia	Other Viral Pneumonia	COVID-19
n	585	93	252	50	190
Age, median [Q1,Q3]	62.0 [51.0,72.0]	60.0 [49.0,70.0]	65.0 [52.0,73.0]	59.5 [52.2,69.8]	61.0 [51.0,70.0]
Ethnicity, n (%)					
Hispanic or Latino	122 (20.9)	12 (12.9)	24 (9.5)	10 (20.0)	76 (40.0)
Not Hispanic or Latino	438 (74.9)	77 (82.8)	218 (86.5)	37 (74.0)	106 (55.8)
Unknown or Not Reported	25 (4.3)	4 (4.3)	10 (4.0)	3 (6.0)	8 (4.2)
Gender, n (%)					
Female	239 (40.9)	46 (49.5)	100 (39.7)	24 (48.0)	69 (36.3)
Male	346 (59.1)	47 (50.5)	152 (60.3)	26 (52.0)	121 (63.7)
Race, n (%) [A]					
Asian	17 (2.9)	3 (3.2)	7 (2.8)	3 (6.0)	4 (2.1)
Black or African American	119 (20.3)	17 (18.3)	55 (21.8)	8 (16.0)	39 (20.5)
Unknown or Not Reported	105 (17.9)	14 (15.1)	33 (13.1)	5 (10.0)	53 (27.9)
White	344 (58.8)	59 (63.4)	157 (62.3)	34 (68.0)	94 (49.5)
Smoking status, n (%)					
Current Smoker	48 (8.2)	9 (9.7)	30 (11.9)	5 (10.0)	4 (2.1)
Never Smoker	230 (39.3)	40 (43.0)	94 (37.3)	22 (44.0)	74 (38.9)
Past Smoker	150 (25.6)	25 (26.9)	78 (31.0)	19 (38.0)	28 (14.7)
Unknown Smoking Status	157 (26.8)	19 (20.4)	50 (19.8)	4 (8.0)	84 (44.2)
BMI, median [Q1,Q3] [B]	28.7 [24.6,34.1]	27.4 [24.6,33.4]	27.0 [22.6,32.7]	26.6 [24.6,31.9]	30.6 [27.2,36.9]
Admit APS score, median [Q1,Q3]	89.0 [64.0,107.0]	90.0 [62.0,105.0]	88.0 [66.0,109.0]	86.0 [64.2,100.0]	90.0 [61.2,106.8]
Admit SOFA score, median [Q1,Q3]	11.0 [8.0,13.0]	11.0 [8.0,14.0]	11.0 [8.0,14.0]	10.0 [7.0,13.0]	11.0 [8.2,13.0]
Cumulative ICU days, median [Q1,Q3] [C]	14.0 [6.0,26.0]	8.0 [4.0,17.0]	10.0 [5.8,20.0]	11.0 [7.5,19.8]	24.0 [14.0,36.8]
Number of ICU stays, median [Q1,Q3]	1.0 [1.0,1.0]	1.0 [1.0,1.0]	1.0 [1.0,2.0]	1.0 [1.0,2.0]	1.0 [1.0,1.0]
Cumulative pred equivalents during admission, median [Q1,Q3]	150.0 [0.0,390.0]	130.0 [0.0,720.0]	114.0 [0.0,300.0]	118.5 [26.5,310.0]	240.0 [26.2,437.5]
Received steroids during admission, n (%)	409 (69.9)	64 (68.8)	160 (63.5)	38 (76.0)	147 (77.4)
Received tocilizumab during admission, n (%)	17 (2.9)	-	1 (0.4)	-	16 (8.4)
Received sarilumab during admission, n (%)	1 (0.2)	-	-	-	1 (0.5)
Sarilumab study drug during admission, n (%)	12 (2.1)	-	-	-	12 (6.3)
Received remdesivir during admission, n (%)	76 (13.0)	-	4 (1.6)	-	72 (37.9)

Remdesivir study drug during admission, n (%)	11 (1.9)	-	-	-	11 (5.8)
Congestive heart failure, n (%)	170 (29.1)	37 (39.8)	89 (35.3)	10 (20.0)	34 (17.9)
Peripheral vascular disease, n (%)	121 (20.7)	19 (20.4)	62 (24.6)	12 (24.0)	28 (14.7)
Cerebrovascular disease, n (%)	123 (21.0)	21 (22.6)	61 (24.2)	14 (28.0)	27 (14.2)
Chronic pulmonary disease, n (%)	209 (35.7)	33 (35.5)	97 (38.5)	21 (42.0)	58 (30.5)
Peptic ulcer disease, n (%)	53 (9.1)	10 (10.8)	25 (9.9)	8 (16.0)	10 (5.3)
Liver disease, n (%)	150 (25.6)	24 (25.8)	76 (30.2)	18 (36.0)	32 (16.8)
Diabetes, n (%)	208 (35.6)	25 (26.9)	85 (33.7)	24 (48.0)	74 (38.9)
Renal disease, n (%)	158 (27.0)	33 (35.5)	77 (30.6)	14 (28.0)	34 (17.9)
Cancer, n (%)	196 (33.5)	42 (45.2)	90 (35.7)	25 (50.0)	39 (20.5)
Immunocompromised flag, n (%)	162 (27.7)	31 (33.3)	78 (31.0)	22 (44.0)	31 (16.3)
Tracheostomy flag, n (%)	151 (25.8)	15 (16.1)	48 (19.0)	7 (14.0)	81 (42.6)
Cumulative intubation days, median [Q1,Q3]	10.0 [4.0,23.0]	5.0 [2.0,12.0]	8.0 [4.0,18.0]	9.0 [3.0,14.0]	21.0 [10.0,35.0]
Discharge disposition, n (%)					
Died [D]	243 (41.5)	37 (39.8)	99 (39.3)	20 (40.0)	87 (45.8)
Home	133 (22.7)	27 (29.0)	49 (19.4)	10 (20.0)	47 (24.7)
LTACH	59 (10.1)	6 (6.5)	27 (10.7)	4 (8.0)	22 (11.6)
Rehab	97 (16.6)	11 (11.8)	48 (19.0)	12 (24.0)	26 (13.7)
SNF	34 (5.8)	6 (6.5)	19 (7.5)	1 (2.0)	8 (4.2)
Hospice	19 (3.2)	6 (6.5)	10 (4.0)	3 (6.0)	-

Supplemental Table 2. **Descriptive features from first day of intubation in patients, excluding patients received in external transfer who were intubated on ICU day 1, grouped by pneumonia category.** Of our cohort of 585 patients, 184 patients (31.4% of the cohort) were received in transfer from another hospital. Of these patients, 139 (75.5%) were intubated at time of transfer or during the first day in our ICU and are excluded from this table.

Feature	Non-Pneumonia Control	Other Pneumonia	Other Viral Pneumonia	COVID-19
n	72	200	36	138
ICU day, median [Q1,Q3]	1.0 [1.0,2.0]	1.0 [1.0,2.0]	1.0 [1.0,2.0]	1.0 [1.0,2.0]
SOFA score, median [Q1,Q3]	12.0 [9.0,14.2]	12.0 [9.0,14.0]	11.5 [7.8,15.0]	11.0 [9.0,13.0]
ECMO flag, n (%)	1 (1.4)	1 (0.5)	-	-
Intubation flag, n (%)	72 (100.0)	200 (100.0)	36 (100.0)	138 (100.0)
Hemodialysis flag, n (%)	3 (4.2)	5 (2.5)	1 (2.8)	-
CRRT flag, n (%)	6 (8.3)	18 (9.0)	5 (13.9)	5 (3.6)
Temperature, median [Q1,Q3]	98.2 [97.6,99.5]	98.3 [97.5,99.4]	98.7 [98.0,99.7]	98.9 [98.3,99.8]
Heart rate, median [Q1,Q3]	88.7 [77.3,107.8]	95.2 [82.0,109.9]	99.8 [86.8,112.9]	85.1 [75.5,96.6]
Systolic blood pressure, median [Q1,Q3]	112.2 [104.3,130.8]	112.8 [104.1,124.7]	111.7 [106.6,124.9]	117.9 [110.6,126.4]
Diastolic blood pressure, median [Q1,Q3]	61.1 [56.1,68.8]	59.8 [55.4,67.7]	59.6 [54.8,65.8]	62.9 [57.4,68.1]
Mean arterial pressure, median [Q1,Q3]	61.0 [56.0,68.0]	60.0 [53.0,65.0]	59.5 [55.0,66.2]	61.0 [57.2,66.0]
Norepinephrine rate, median [Q1,Q3]	0.2 [0.1,0.3]	0.2 [0.1,0.3]	0.2 [0.1,0.2]	0.1 [0.1,0.2]
Norepinephrine flag, n (%)	46 (63.9)	126 (63.0)	20 (55.6)	98 (71.0)
Respiratory rate, median [Q1,Q3]	22.6 [19.3,26.5]	22.3 [19.8,26.1]	23.7 [20.1,27.3]	25.0 [22.0,28.1]
Oxygen saturation, median [Q1,Q3]	96.5 [94.9,98.1]	96.7 [95.2,98.0]	96.6 [94.9,98.7]	94.8 [93.6,96.1]
Urine output, median [Q1,Q3]	857.5 [461.2,1558.8]	647.5 [244.0,1293.2]	800.0 [190.0,1237.5]	900.0 [483.0,1400.0]
GCS eye opening, median [Q1,Q3]	1.0 [1.0,3.0]	1.0 [1.0,3.0]	1.0 [1.0,2.0]	1.0 [1.0,3.0]
GCS motor response, median [Q1,Q3]	4.0 [1.0,5.0]	4.0 [1.0,5.0]	4.0 [1.0,4.0]	2.0 [1.0,5.0]
GCS verbal response, median [Q1,Q3]	1.0 [1.0,1.0]	1.0 [1.0,1.0]	1.0 [1.0,1.0]	1.0 [1.0,1.0]
RASS score, median [Q1,Q3]	-2.0 [-4.0,-1.0]	-2.0 [-3.5,-1.0]	-3.0 [-4.0,-2.0]	-3.0 [-4.0,-2.0]
PEEP, median [Q1,Q3]	5.0 [5.0,8.1]	5.0 [5.0,8.9]	5.0 [5.0,7.8]	11.0 [10.0,14.0]
FiO2, median [Q1,Q3]	50.0 [45.0,70.5]	59.0 [45.0,75.0]	58.8 [46.7,70.0]	73.3 [60.6,85.0]
Plateau Pressure, median [Q1,Q3]	22.4 [18.8,25.6]	21.5 [17.5,26.3]	20.8 [16.8,23.2]	24.5 [21.8,28.7]
Lung Compliance, median [Q1,Q3]	31.0 [21.5,38.0]	33.5 [25.0,40.5]	30.0 [25.2,40.8]	34.5 [25.8,43.7]
PEEP changes, median [Q1,Q3]	1.0 [1.0,2.0]	1.0 [1.0,2.0]	1.0 [1.0,2.0]	2.0 [1.0,2.0]
Respiratory rate changes, median [Q1,Q3]	2.0 [1.0,3.0]	2.0 [1.0,2.0]	2.0 [1.0,2.0]	2.0 [1.0,2.0]
FiO2 changes, median [Q1,Q3]	2.0 [1.8,3.0]	2.0 [2.0,3.0]	2.0 [2.0,3.0]	2.0 [2.0,3.0]
ABG pH, median [Q1,Q3]	7.4 [7.3,7.4]	7.3 [7.3,7.4]	7.3 [7.3,7.4]	7.4 [7.3,7.4]
ABG PaCO2, median [Q1,Q3]	37.7 [30.8,42.0]	39.5 [32.9,46.2]	37.0 [33.9,47.7]	41.0 [37.0,45.0]
ABG PaO2, median [Q1,Q3]	103.8 [85.1,126.4]	106.1 [86.1,131.5]	108.7 [85.0,125.0]	99.1 [84.8,116.2]
PaO2FIO2 ratio, median [Q1,Q3]	140.0 [91.0,237.1]	136.0 [90.0,200.2]	129.5 [99.5,228.2]	98.6 [71.1,126.6]
WBC count, median [Q1,Q3]	10.7 [7.0,19.2]	13.0 [8.0,17.0]	9.6 [6.7,14.8]	9.8 [6.7,12.5]

Lymphocytes, median [Q1,Q3]	0.5 [0.3,1.1]	0.9 [0.5,1.6]	0.8 [0.4,1.6]	0.8 [0.6,1.3]
Neutrophils, median [Q1,Q3]	7.6 [3.4,14.1]	9.7 [5.2,14.4]	7.8 [3.3,12.7]	7.8 [5.4,10.4]
Hemoglobin, median [Q1,Q3]	9.8 [8.1,11.1]	9.8 [8.2,11.8]	8.4 [7.5,10.4]	12.1 [10.9,13.2]
Platelets, median [Q1,Q3]	129.0 [54.5,223.0]	184.0 [103.5,269.8]	196.0 [61.5,274.5]	215.5 [155.8,304.2]
Bicarbonate, median [Q1,Q3]	23.0 [19.7,26.1]	22.2 [19.0,25.0]	23.2 [20.4,25.0]	23.0 [21.0,26.0]
Creatinine, median [Q1,Q3]	1.3 [0.9,2.0]	1.4 [0.8,1.9]	1.1 [0.7,1.7]	0.9 [0.8,1.4]
Albumin, median [Q1,Q3]	3.0 [2.6,3.5]	3.1 [2.7,3.6]	2.6 [2.3,3.1]	3.3 [3.0,3.5]
Bilirubin, median [Q1,Q3]	0.9 [0.6,1.7]	0.8 [0.6,1.5]	0.7 [0.5,1.0]	0.6 [0.5,0.8]
CRP, median [Q1,Q3]	137.8 [57.0,281.0]	54.2 [8.2,95.3]	-	165.0 [93.2,243.3]
D dimer, median [Q1,Q3]	1382.0 [468.0,3222.0]	2230.5 [483.2,6851.1]	1564.0 [876.0,1823.0]	570.0 [290.2,2086.5]
Ferritin, median [Q1,Q3]	387.8 [159.5,3622.1]	535.5 [105.5,1143.9]	746.5 [465.7,1027.2]	729.7 [423.8,1178.0]
LDH, median [Q1,Q3]	378.0 [248.0,532.8]	332.0 [251.5,541.2]	299.0 [187.5,330.0]	454.0 [347.2,583.5]
Lactic acid, median [Q1,Q3]	2.2 [1.4,3.0]	1.9 [1.3,3.1]	1.7 [1.3,2.0]	1.4 [1.2,1.9]
Procalcitonin, median [Q1,Q3]	0.7 [0.1,2.3]	0.6 [0.1,2.3]	1.1 [0.2,9.6]	0.3 [0.2,1.0]

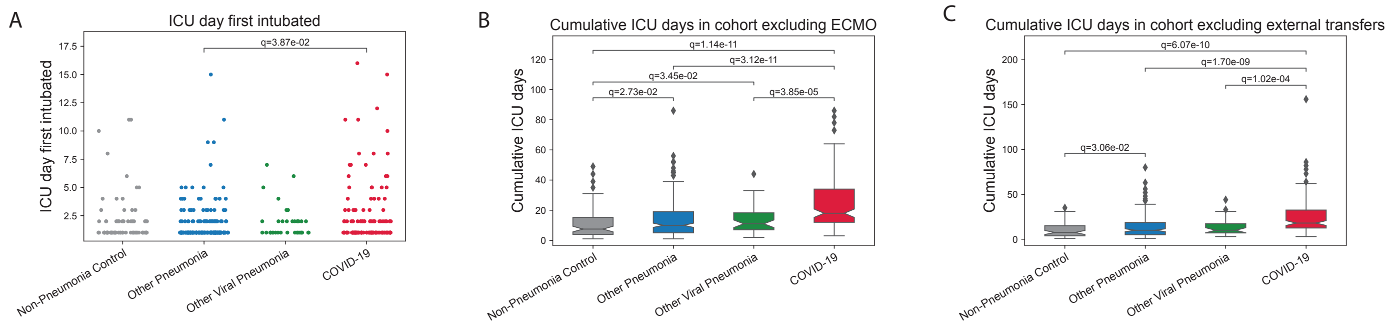

Supplemental Figure 1. Additional demographics and outcomes of the cohort, grouped by pneumonia category. (**A**) ICU day of first intubation, excluding patients received in external transfer (total patients received in external transfer: 184 patients, 31.4% of the cohort), who were intubated on ICU day 1 at our hospital. 139 patients (75.5%) of the patients received in external transfer were intubated at the time of transfer or during the first day in our ICU. (**B**) Cumulative ICU days excluding patients who received ECMO support. Data include only days at our hospital and do not capture ICU LOS from a transferring hospital. (**C**) Cumulative ICU days excluding patients received in external transfer. Box-and-whisker plots: box shows quartiles and median, whiskers show minimum and maximum except for outliers, which are shown as individual data points. Notches are bootstrapped 95% confidence interval of median. Numerical values were compared using Mann-Whitney U tests with false-discovery rate (FDR) correction using the Benjamini-Hochberg procedure. A q-value < 0.05 was our threshold for statistical significance.

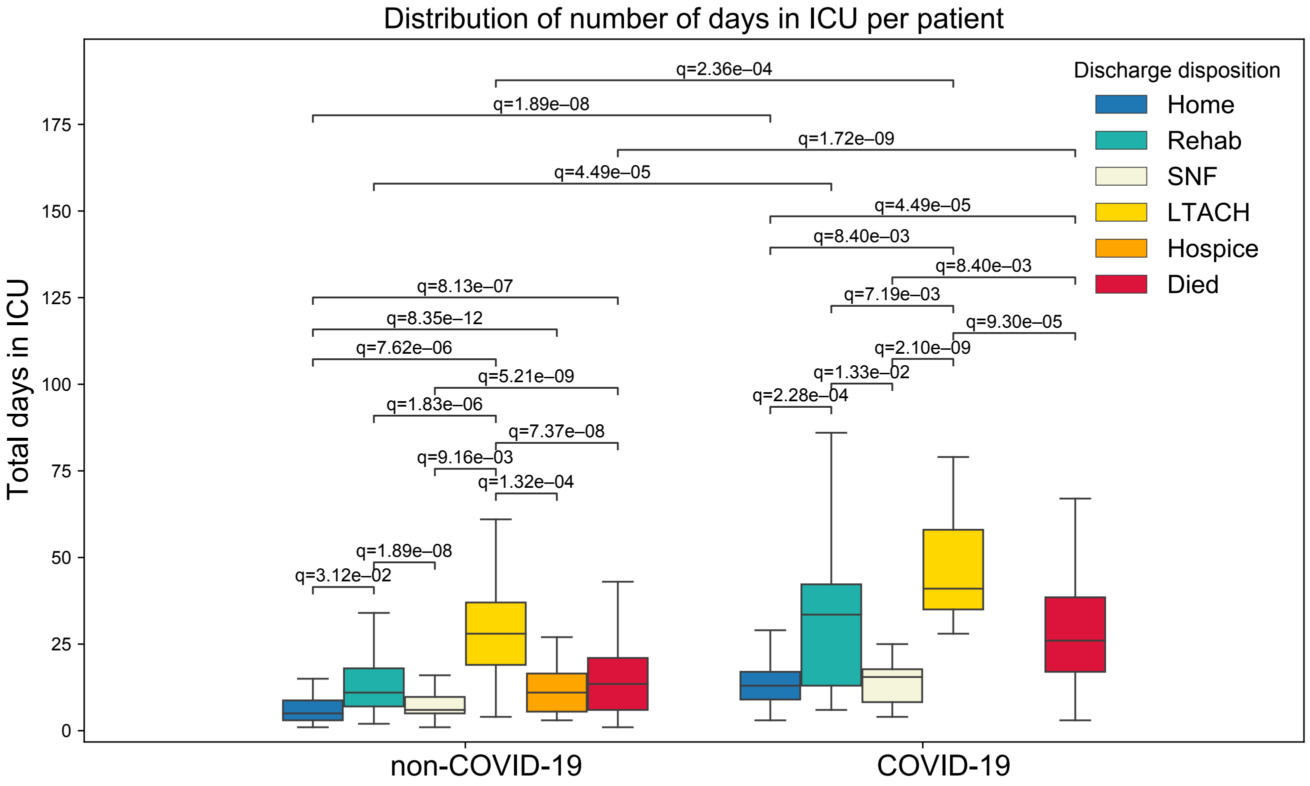

Supplemental Figure 2. Length of stay by discharge disposition among patients with and without COVID-19. Patients with severe SARS-CoV-2 pneumonia experienced significantly longer ICU LOS for all discharge disposition groups, except Skilled Nursing Facility (SNF; not significantly different) and Hospice (no patients in COVID-19 group). No patient was transferred back to their referring center intubated. Box-and-whisker plots: box shows quartiles and median, whiskers show minimum and maximum except for outliers, which are shown as individual data points. Numerical values were compared using Mann-Whitney U tests with false-discovery rate (FDR) correction using the Benjamini-Hochberg procedure. A q-value < 0.05 was our threshold for statistical significance.

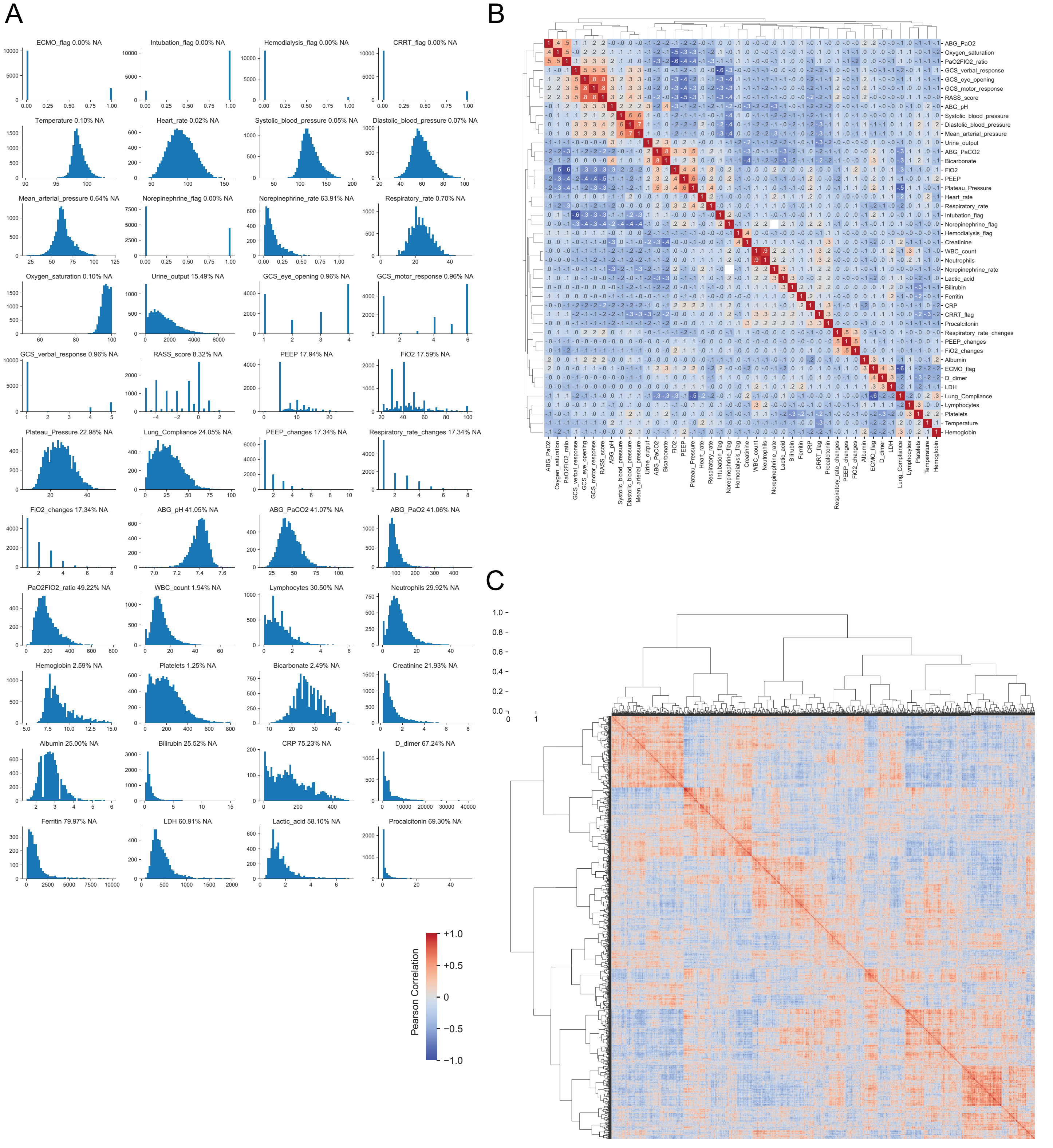

Supplemental Figure 3. Clinical parameters and their correlation. (**A**) Histogram display of 44 clinical values extracted from the EHR. (**B**) Pearson correlation matrix of clinical parameters; some measurements in our dataset displayed a high correlation due to mathematical or physiological coupling (e.g., plateau pressure, PEEP and lung compliance; $PaCO_2$ and bicarbonate). (**C**) Pearson correlation between different patient-days as used as an intermediate step for the “Similarity” clustering strategy.

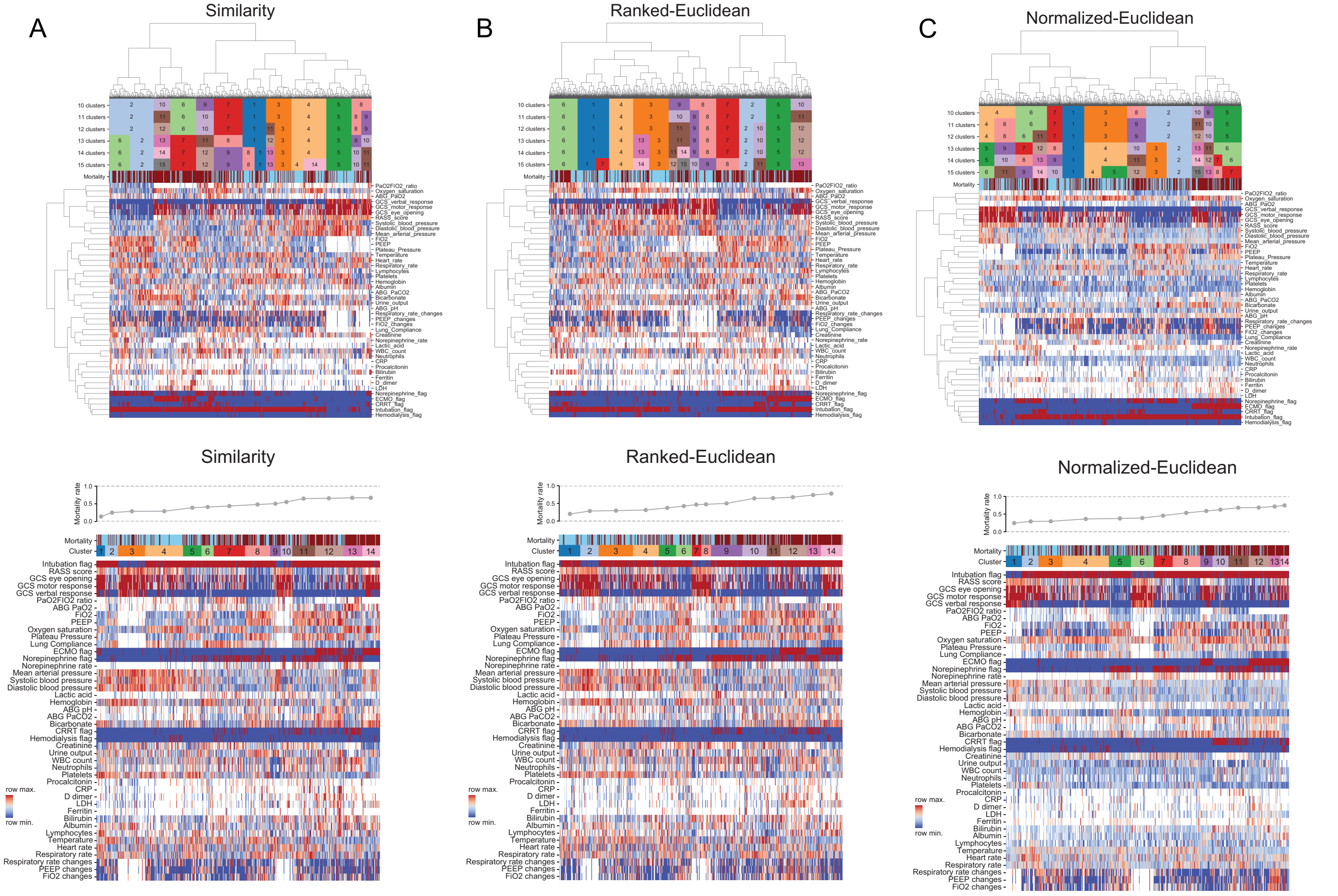

Supplemental Figure 4. Comparing three different clustering strategies. (**A**) Similarity, (**B**) Ranked-Euclidean, and (**C**) Normalized-Euclidean strategies. For each method, hierarchical clustering of clinical parameters (rows) and columns (patient-days) is shown on the top, grouping patient-days into 10-15 separate clusters. The bottom panels show re-ordered clustering with columns organized into clusters and sorted by ascending cluster mortality and rows organized into physiologically similar groups. Cluster mortality is shown above the heatmaps.

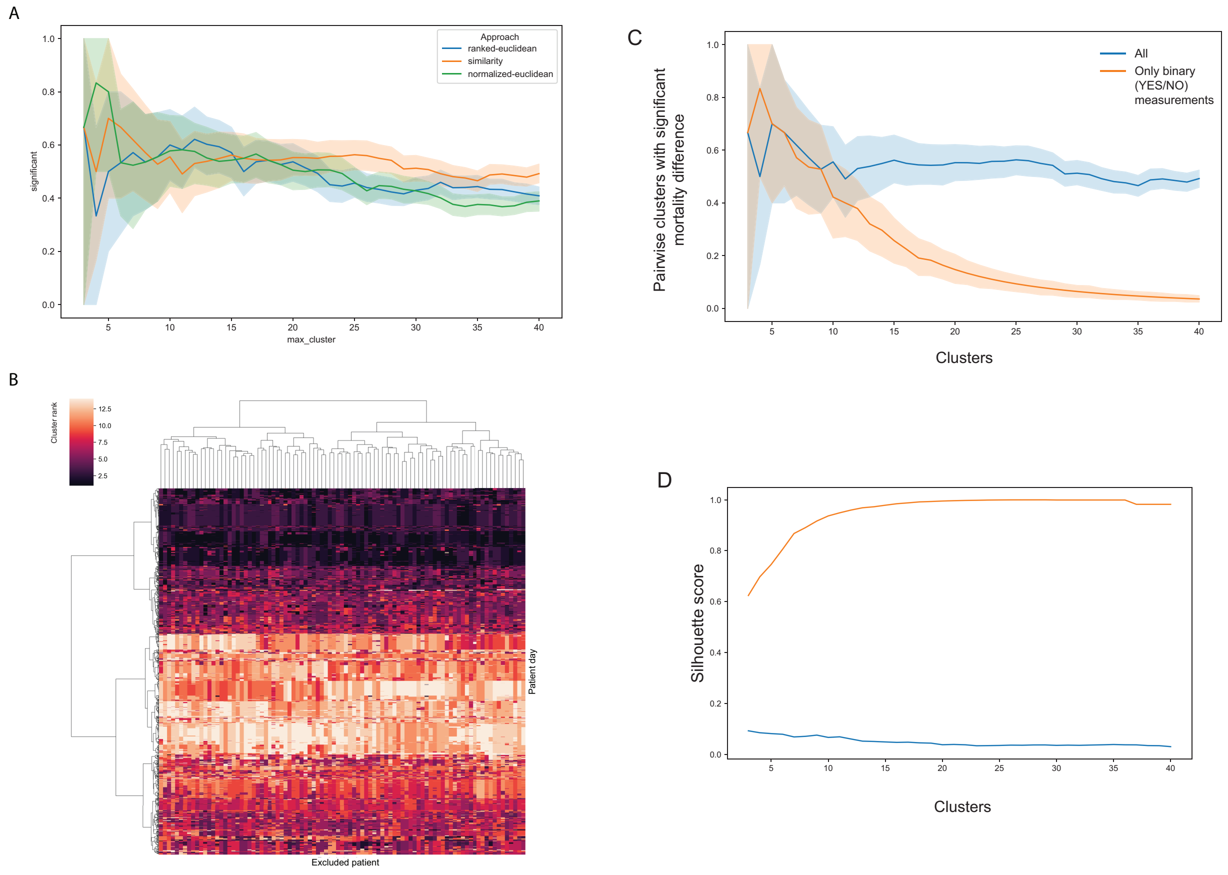

Supplemental Figure 5. Cluster mortality differentiation and robustness against small data perturbations. (**A**) Fraction of all possible pairs between two clusters that show a significantly different mortality at p < 0.01. X-axis shows different cutoffs for total number of clusters. Shaded area is the bootstrapped 95th percentile. (**B**) Allocation of individual patient-days (rows) to clusters following 100 randomizations, in which a single patient hospitalization has been excluded (columns). Cluster rank is the rank of cluster based on associated mortality with 1 being lowest and 14 being highest. (**C**) Clustering as described in panel (A) using “Similarity” approach but only using binary YES/NO flags. Evaluation of performance by share of pairwise clusters that show significantly different mortality at p < 0.01 as in panel (A). (**D**) Corresponding Silhouette scores for the methods in (C). Note that despite YES/NO flags reaching higher compactness of clusters as indicated by higher Silhouette scores, the clinically-relevant ability to distinguish clusters based on mortality is not increased as seen in (C).

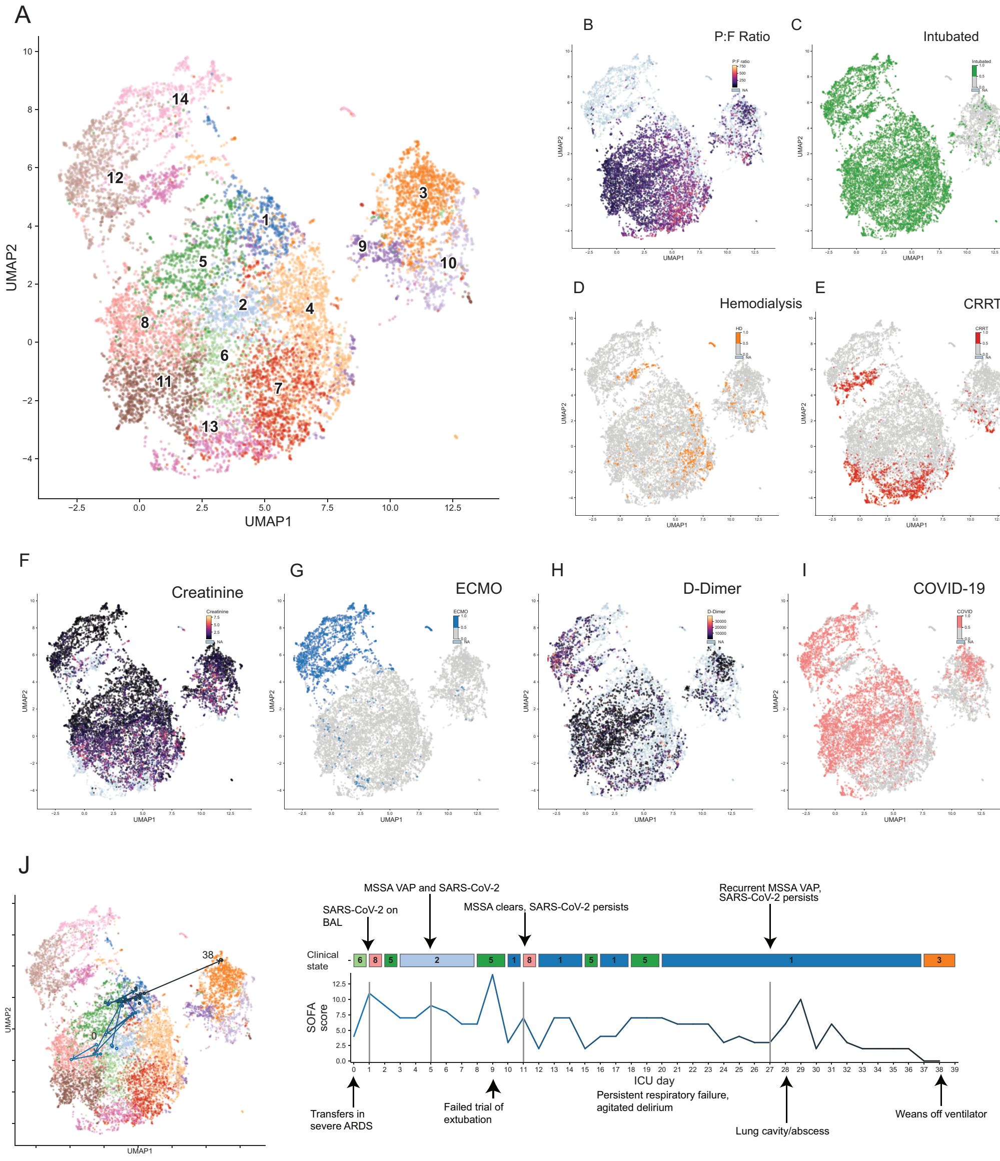

Supplemental Figure 6. UMAP and feature plots. (**A**) UMAP with colors and numbers representing *CarpeDiem*-defined clusters (clinical states). (**B-H**) Feature plots for individual parameters. (**I**) Patient-days from patients with COVID-19. (**J**) Example trajectory of a patient who was liberated from mechanical ventilation and was discharged home. A UMAP on the left demonstrating clinical transitions is labeled with the starting clinical state (day 0) and the day of discharge from the ICU (day 38). On the right, microbiological and clinical events overlaid on clinical state transitions are shown. Vertical lines indicate timepoints of BAL sampling. The beginning of the ICU course is outlined in light blue, whereas the end is dark blue, on both the timeline and the UMAP. More example trajectories are available in an interactive web app available at https://nupulmonary.org/carpediem. HFNC = high-flow nasal cannula, ECMO = extracorporeal membrane oxygenation, CRRT = continuous renal replacement therapy, MSSA = methicillin-sensitive *Staphylococcus aureus*.

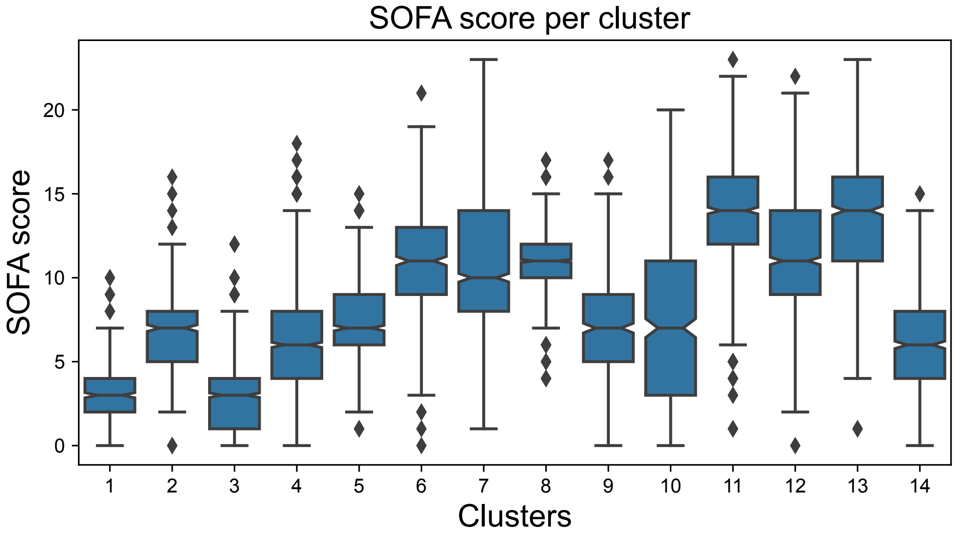

Supplemental Figure 7. **SOFA scores per cluster.** Median [IQR] SOFA scores were calculated for the days represented in each cluster. Box-and-whisker plots: box shows quartiles and median, whiskers show minimum and maximum except for outliers, which are shown as individual data points. Notches are bootstrapped 95% confidence interval of median.

A

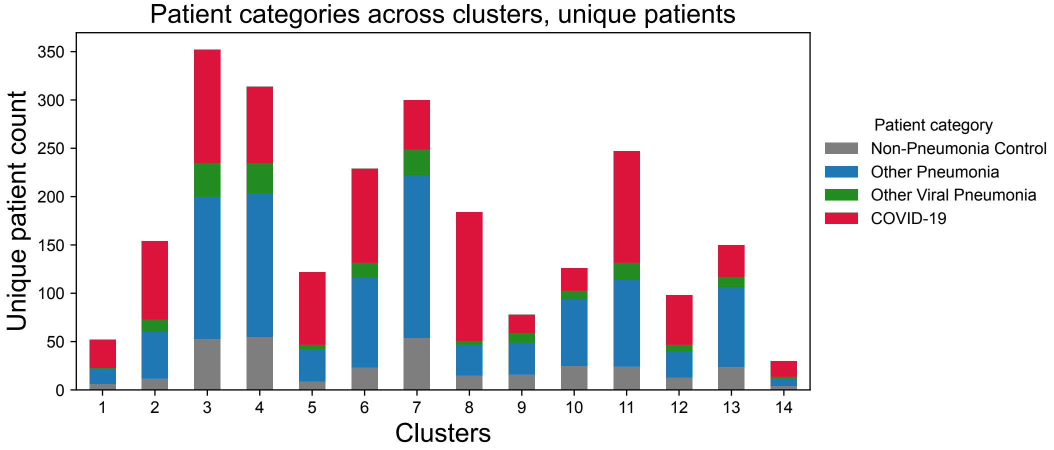

B

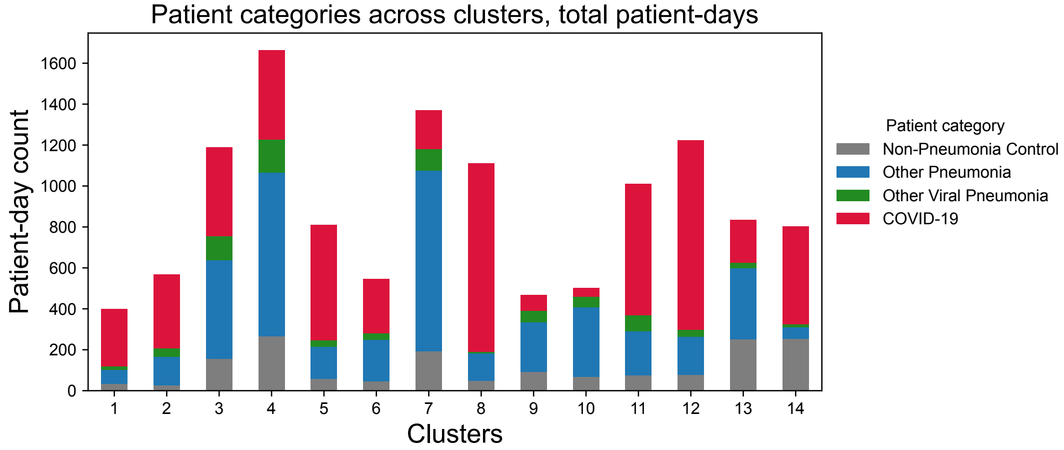

Supplemental Figure 8. Every cluster is comprised of patients from all four pneumonia categories. (**A**) Count of unique patients that contribute to clusters, colored by category. (**B**) Patient-days, colored by category.

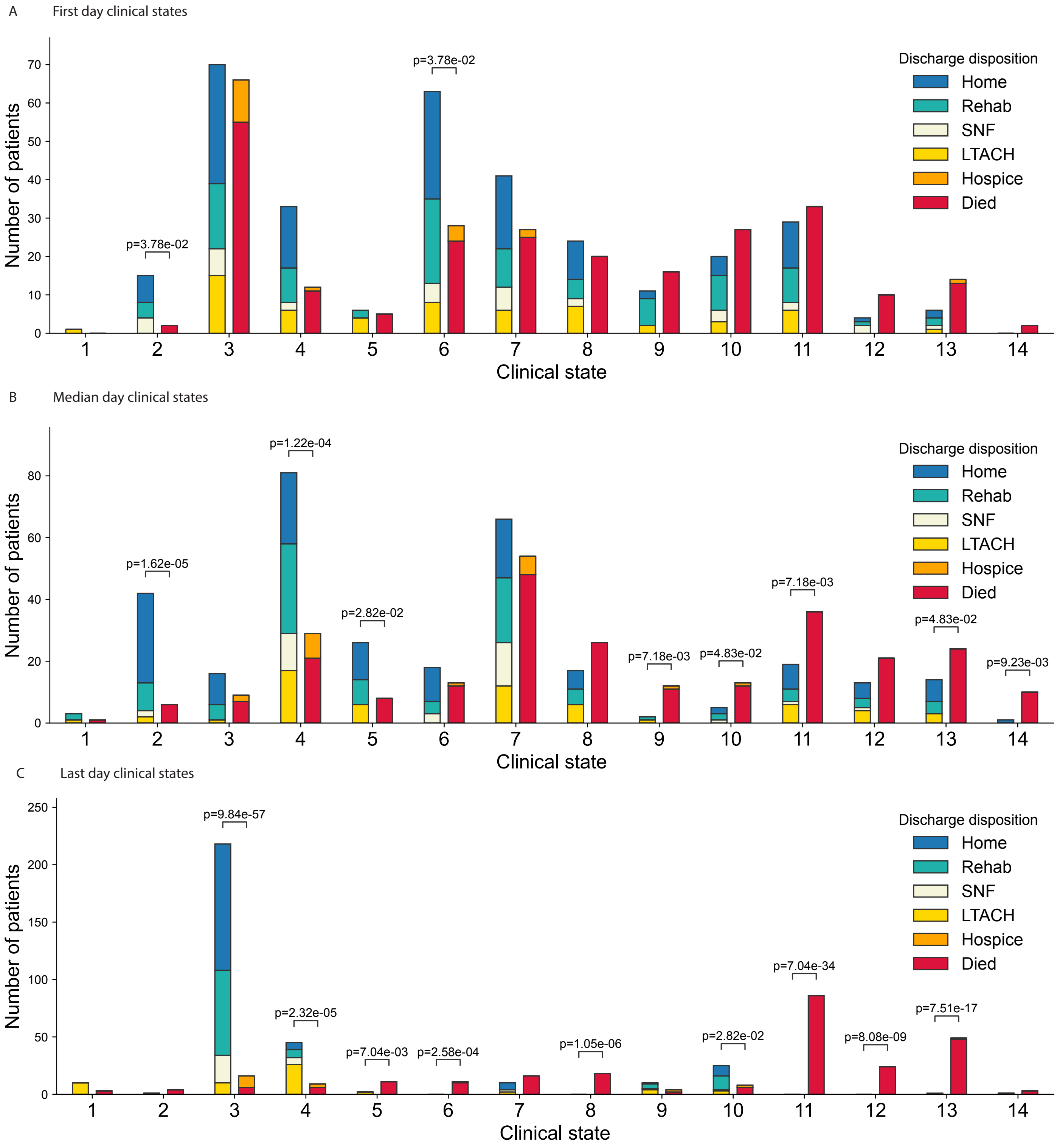

Supplemental Figure 9. Clinical states are associated with outcome. Association between the clinical state occupied by each patient on their first (**A**), median (**B**), or last (**C**) ICU day and their discharge disposition. Outcomes are displayed in two columns: the first column aggregates favorable discharge dispositions (Home, Rehab, SNF, LTACH), the second column aggregates unfavorable discharge dispositions (Hospice, Died). SNF = Skilled Nursing Facility, LTACH = long-term acute care hospital.

Categorical values were compared using Fisher's Exact tests with FDR correction using the Benjamini-Hochberg procedure. A q-value < 0.05 was our threshold for statistical significance.

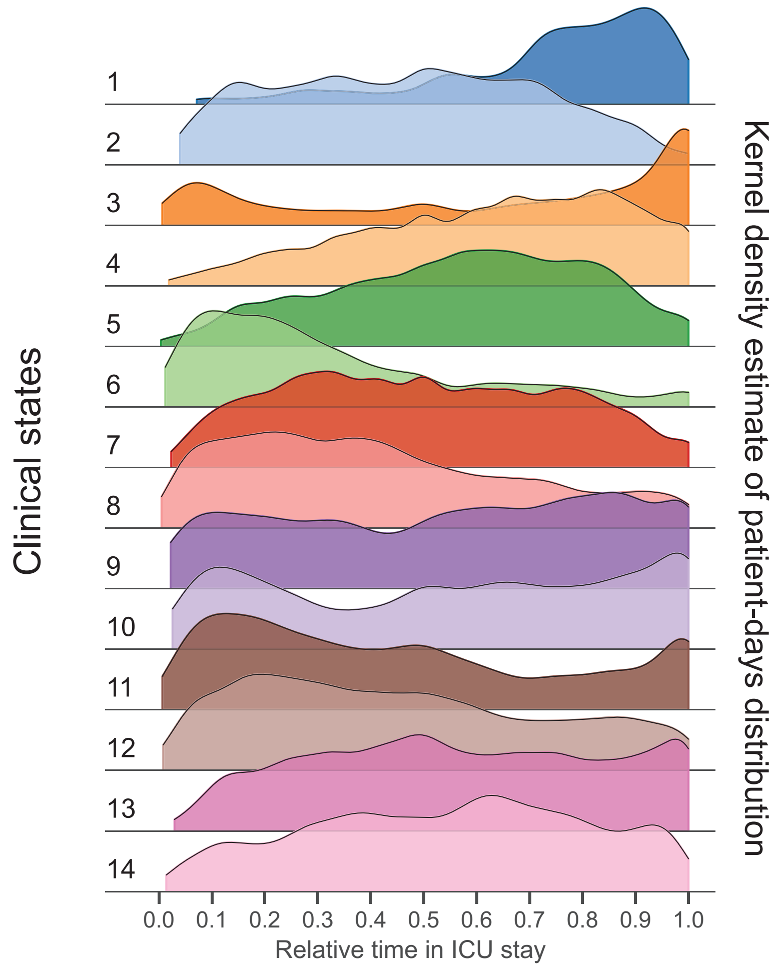

Supplemental Figure 10. Kernel density estimate plots showing relative time through the ICU stay for each clinical state.

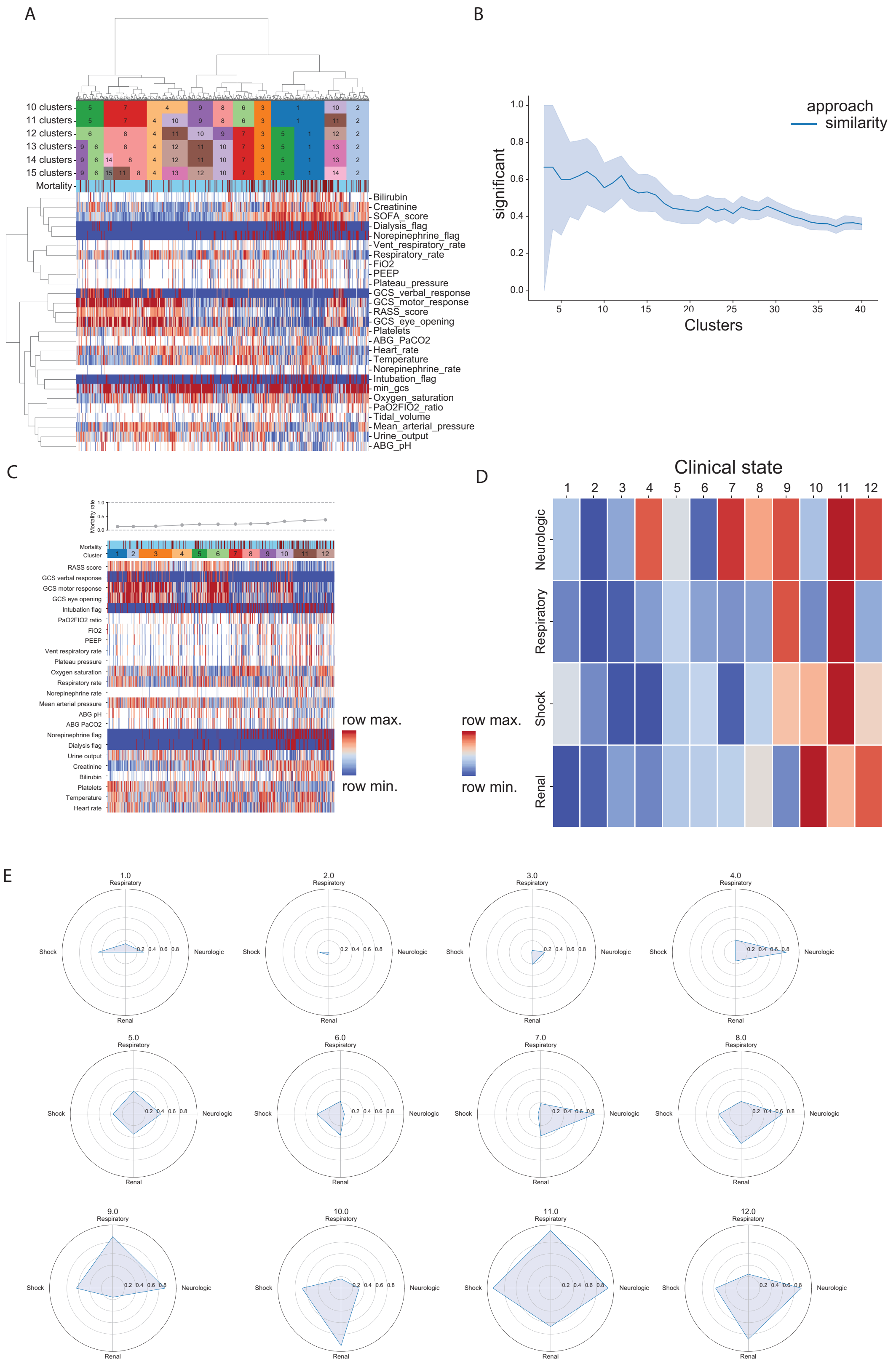

Supplemental Figure 11. Application of *CarpeDiem* to the MIMIC-IV dataset. (**A**) Hierarchical clustering of 27 clinical parameters (rows) with columns representing 15,642 ICU patient-days from 1,284 patients. (**B**) The fraction of pairs between clusters that show a significantly different mortality rate at different numbers of clusters. Shaded area is bootstrapped 95% confidence interval. (**C**) Heatmap of data from (A) re-ordered from lowest to highest mortality, using 12 clusters. The top strip signifies hospital mortality of the patient shown in the column (blue = survived, red = died). The hospital mortality rate associated with each cluster is shown above the heatmap.

(**D**) Heatmap of the composite signal from each cluster and physiologic group with ordering same as (C). (**E**) Spider plots of normalized composite features from (D) for each clinical state.

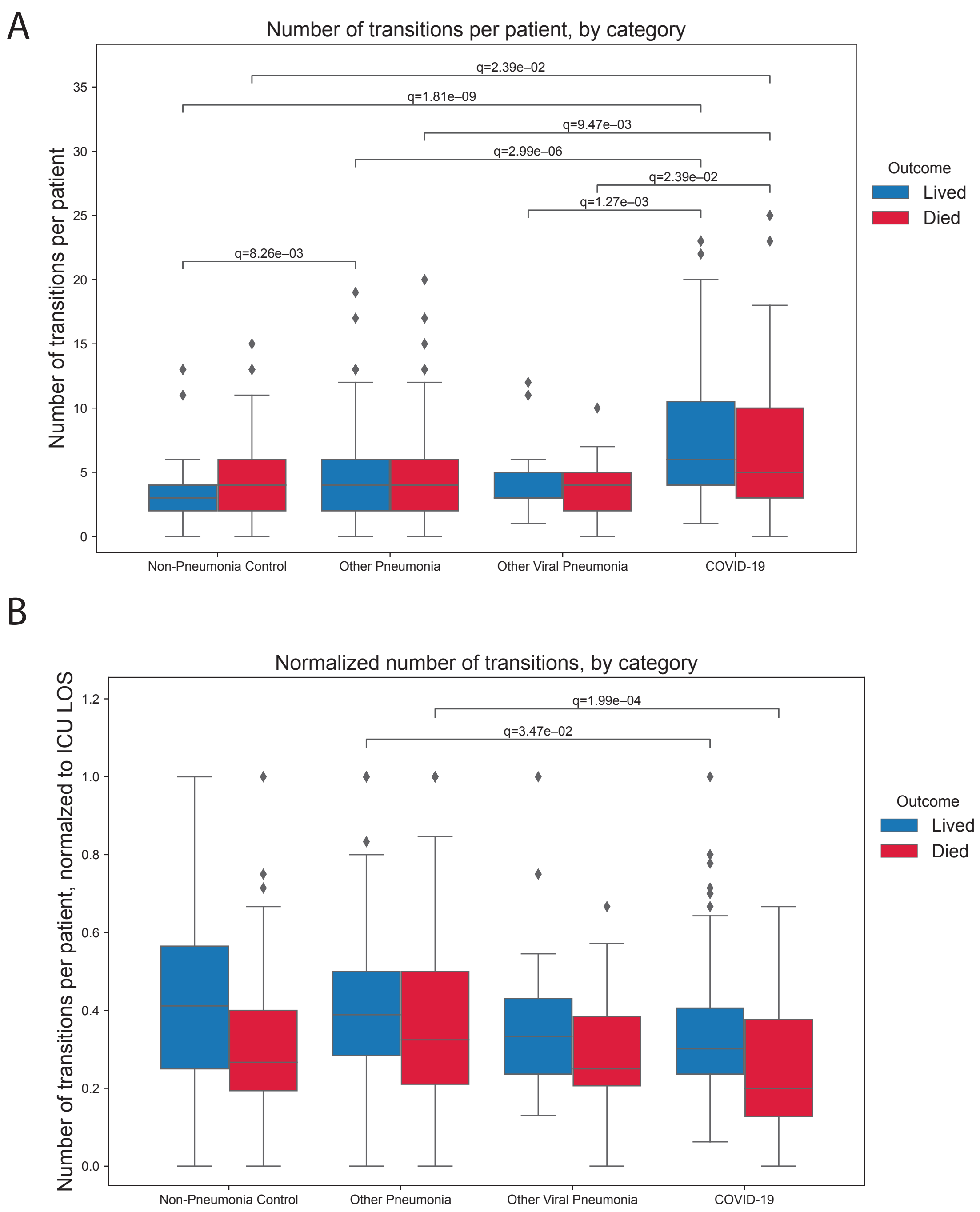

Supplemental Figure 12. Patients with COVID-19 have a higher absolute number of transitions but fewer when normalized for their longer ICU LOS. (**A**) Distribution of transitions per patient. (**B**) Distribution of transitions normalized by ICU LOS. Box-and-whisker plots: box shows quartiles and median, whiskers show minimum and maximum except for outliers, which are shown as individual data points. Numerical values were compared using Mann-Whitney U tests with false-discovery rate (FDR) correction using the Benjamini-Hochberg procedure. A q-value < 0.05 was our threshold for statistical significance.

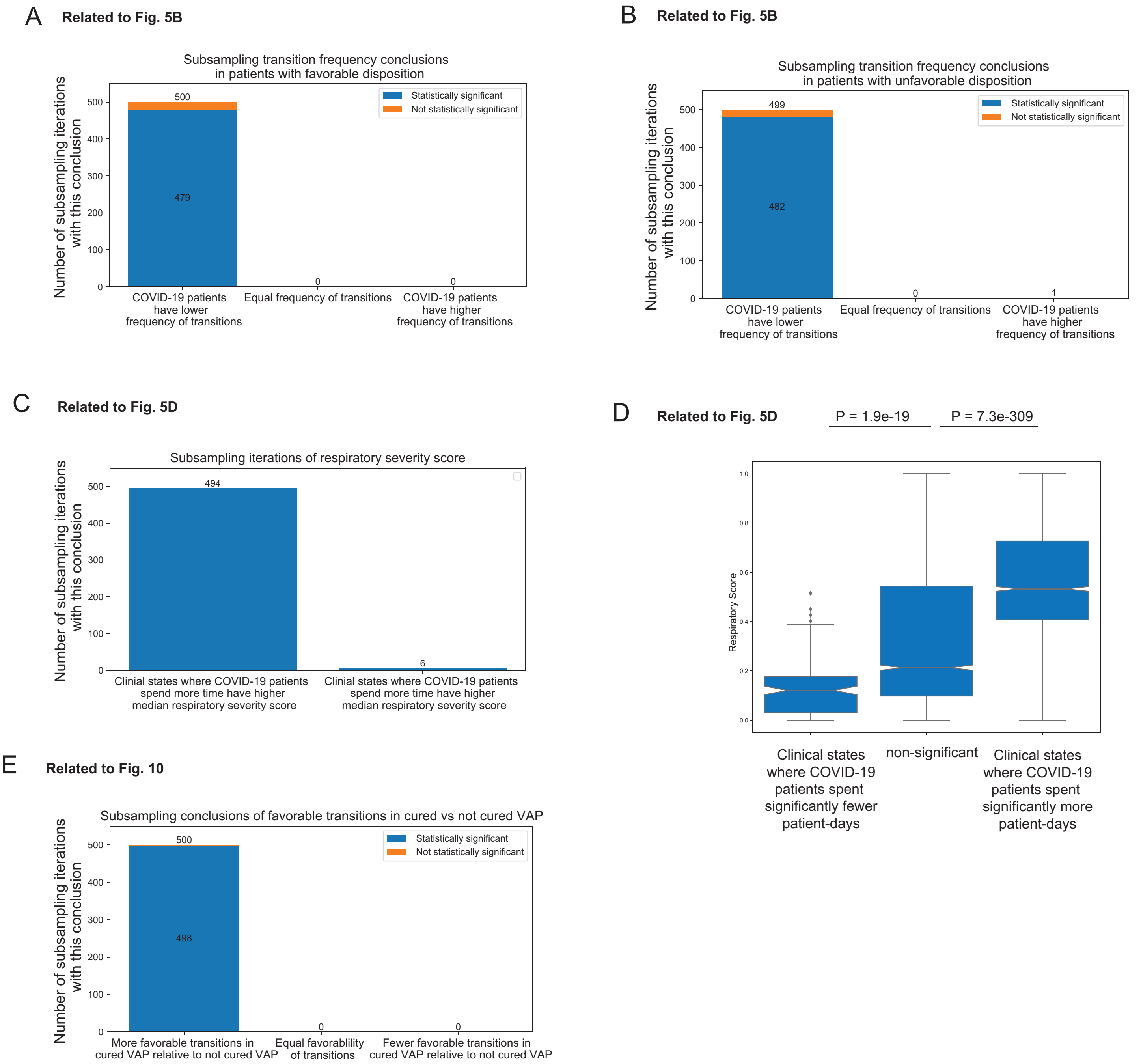

Supplemental Figure 13. Conclusions drawn from the full dataset are robust to random subsampling of 80% of patients over 500 iterations. As pertaining to Figure 5B, the frequency of transitions between clinical states was significantly lower in patients with COVID-19 compared with other patients, both in patients who had a favorable discharge disposition (**A**) and in patients who had an unfavorable discharge disposition (**B**). Patients with COVID-19 had a lower normalized frequency of transitions in 500 of 500 iterations among patients who had a favorable disposition (479 were statistically significant) and in 499 of 500 iterations among patients who had an unfavorable disposition (482 were statistically significant). (**C**) As pertaining to Figure 5D, we found that clinical states enriched in patients with COVID-19 had higher median respiratory severity scores (494 of 500 subsampling iterations). (**D**) Distribution of respiratory severity scores for clusters in which LOS is significantly shorter among patients with COVID-19, in which clusters have no significant difference in LOS, and in which LOS is significantly longer among patients with COVID-19. (**E**) As pertaining to Figure 10A, 499 of 500 subsampling iterations found that there were more favorable transitions in episodes of cured VAP compared to episodes of VAP that were not cured. In all panels, significance indicates two-sided Mann-Whitney U test p <

0.05. Box-and-whisker plots: box shows quartiles and median, whiskers show minimum and maximum except for outliers, which are shown as individual data points. Notches are bootstrapped 95% confidence interval of median.

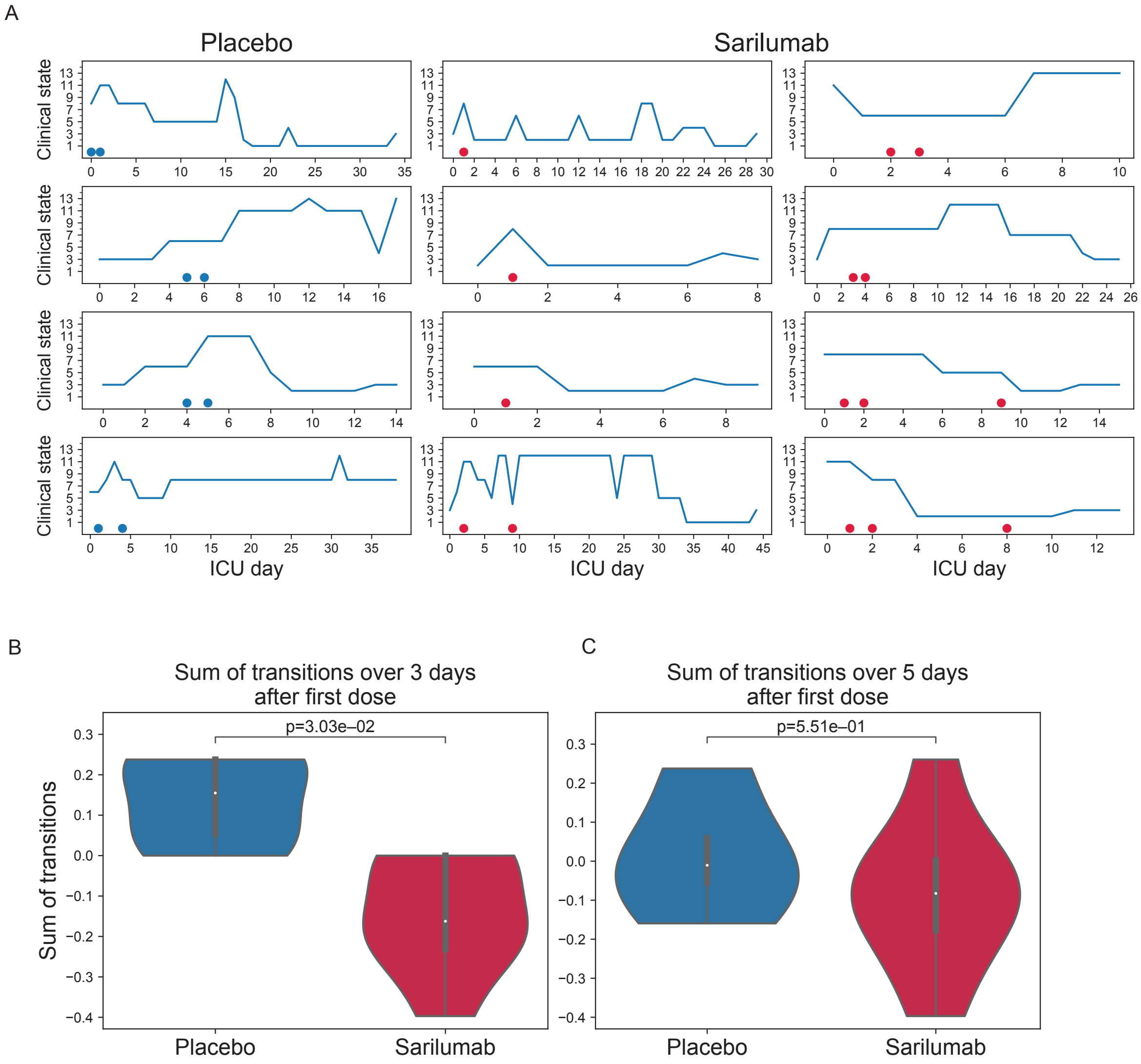

Supplemental Figure 14. *CarpeDiem* provides potential insights in the subset of patients co-enrolled in a randomized placebo-controlled trial of sarilumab for COVID-19 and respiratory failure. (**A**) Timeline of patients who received placebo (first column) or sarilumab (second and third columns) with x-axis showing ICU day and y-axis showing *CarpeDiem* clinical state. Dots indicate day of placebo or sarilumab administration. (**B**) Sum of transitions grouped by placebo versus sarilumab for the three days following the first dose. Higher sums of transitions reflect transitions to unfavorable (higher mortality) clusters. (**C**) Sum of transitions grouped by placebo versus sarilumab for the five days following the first dose.

Violin plots: colored area shows the kernel density estimate of the values distribution; inside, box-and-whisker plots are drawn in black with white dot corresponding to the median. Numerical values were compared by Mann-Whitney U tests with false-discovery rate (FDR) correction using the Benjamini-Hochberg procedure. A q-value < 0.05 was our threshold for statistical significance.

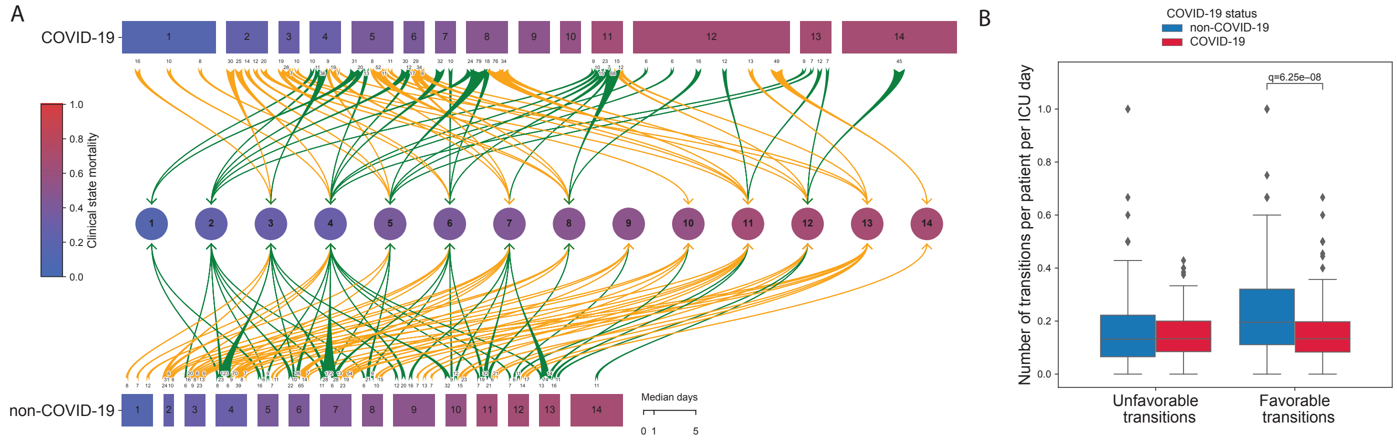

Supplemental Figure 15. Patients with SARS-CoV-2 pneumonia have a longer length of stay and fewer transitions between clinical states per day compared to patients with non-COVID-19-related respiratory failure. (**A**) Clinical states are ordered and numbered 1-14 by their associated mortality rate (blue to red). Rectangle width reflects median days per clinical state. Transitions marked by green arrows are to a more favorable (lower mortality) clinical state; yellow arrows mark transitions to a less favorable (higher mortality) clinical state. Numbers at the arrow bases represent the number of transitions between the two clinical states connected by the arrow. Only transitions that occurred more than five times are shown (cutoff of 30 transitions used in Figure 6). (**B**) Quantification of the number of transitions per patient per ICU day, grouped by COVID-19 and outcome favorability.

Box-and-whisker plots: box shows quartiles and median, whiskers show minimum and maximum except for outliers, which are shown as individual data points. Numerical values were compared using Mann-Whitney U tests with false-discovery rate (FDR) correction using the Benjamini-Hochberg procedure. A q-value < 0.05 was our threshold for statistical significance.

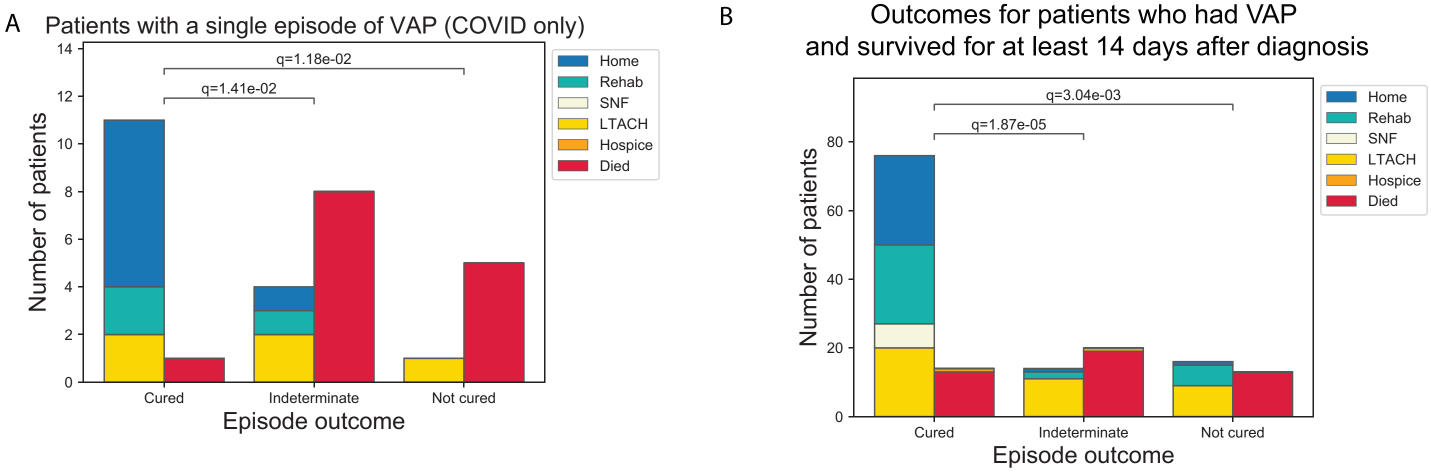

Supplemental Figure 16. Unresolving VAP is associated with worse outcomes. (**A**) Mortality associated with a single episode of VAP among patients with COVID-19. (**B**) Outcomes for patients who did not die within 14 days following the onset of their VAP episode. Outcomes are displayed in two columns: the first column aggregates favorable discharge dispositions (Home, Rehab, SNF, LTACH), the second column aggregates unfavorable discharges (Hospice, Died).

Categorical values were compared using Fisher's Exact tests with FDR correction using the Benjamini-Hochberg procedure. A q-value < 0.05 was our threshold for statistical significance.

A

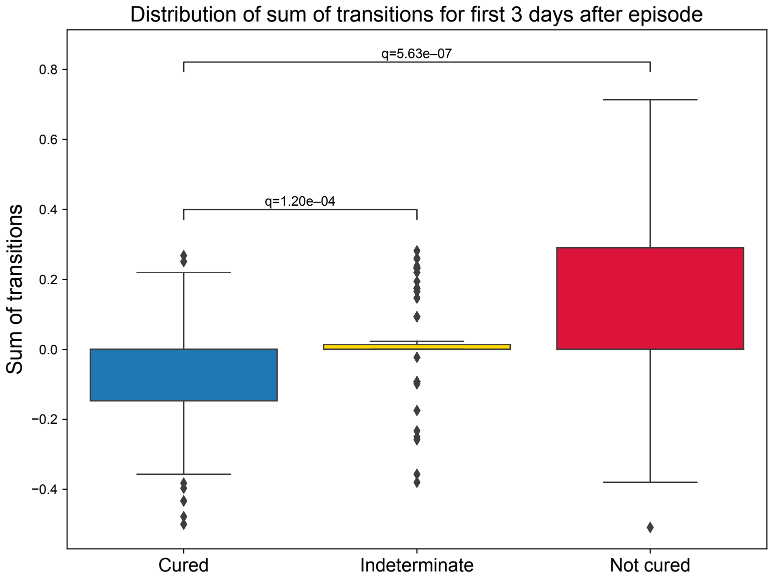

Supplemental Figure 17. Differences in the summative favorability of transitions is evident as early as day 3 following the diagnosis of VAP. (**A**) Sum of transitions grouped by VAP episode outcome for the three days following the diagnosis of VAP. Higher sums of transitions reflect transitions to unfavorable (higher mortality) clusters. Box-and-whisker plots: box shows quartiles and median, whiskers show minimum and maximum except for outliers, which are shown as individual data points. Numerical values were compared using Mann-Whitney U tests with false-discovery rate (FDR) correction using the Benjamini-Hochberg procedure. A q-value < 0.05 was our threshold for statistical significance.

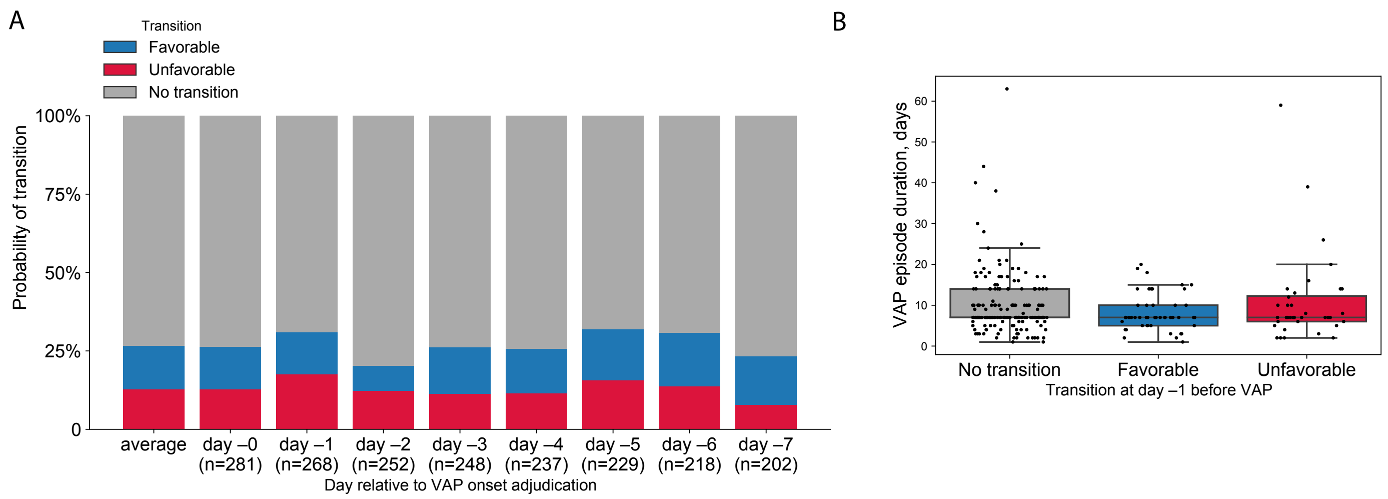

Supplemental Figure 18. Clinical state favorability changes before VAP diagnosis. (**A**) An increase in unfavorable transitions occurs a day before VAP diagnosis (day **-1**). (**B**) Day -1 transitions are not associated with duration of the ensuing VAP episode.
Box-and-whisker plots: box shows quartiles and median, whiskers show minimum and maximum except for outliers, which are shown as individual data points. Numerical values were compared using Mann-Whitney U tests with false-discovery rate (FDR) correction using the Benjamini-Hochberg procedure. A q-value < 0.05 was our threshold for statistical significance.

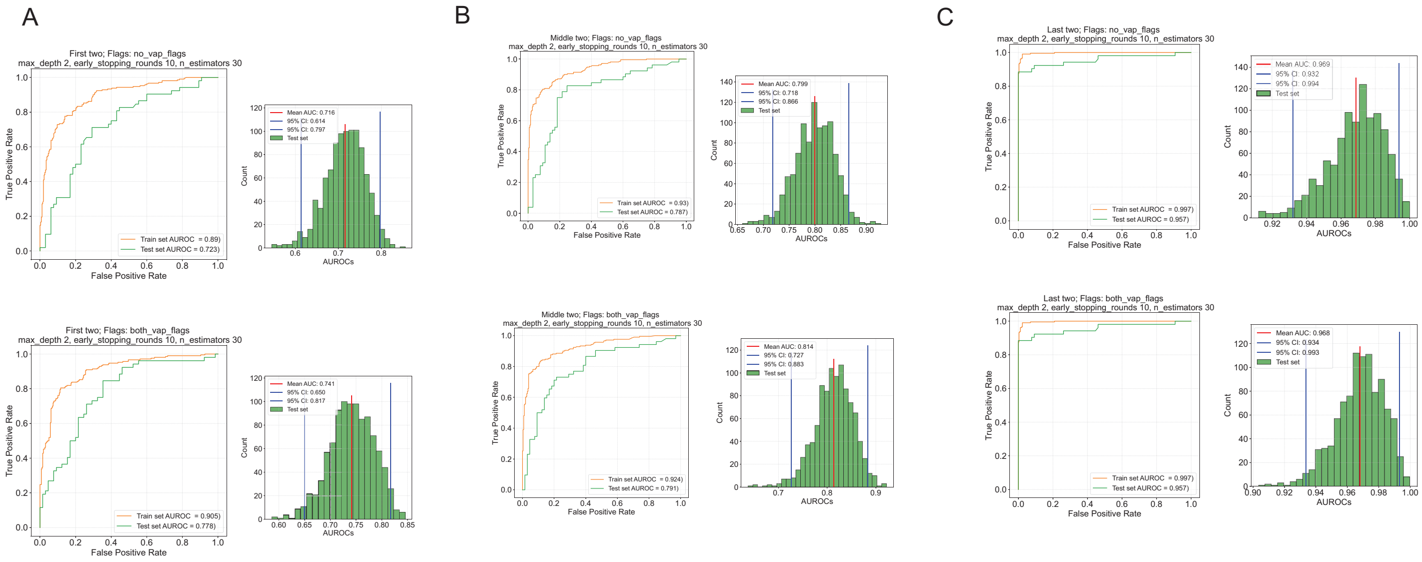

Supplemental Figure 19. Gradient boosting modeling reveals minimal increase in predictive capability when VAP and VAP cure status are added to clinical parameters measured at the beginning, in the middle, and at the end of the ICU course. Area under the receiver operating characteristics (AUROC) curve values for clinical parameters in the gradient boosting analysis predicting unfavorable hospital outcome, with corresponding confidence interval plots obtained using bootstrapping. Using the worst features from the (**A**) first two days, (**B**) median two days, and (**C**) last two days. Bottom row has addition of two flags, *had_vap* and *vap_interdeterminate_uncured*, to indicate a diagnosis of VAP during the ICU stay and a VAP outcome other than cured.
