# Supplementary material for: Machine learning links unresolving secondary pneumonia to mortality in patients with severe pneumonia, including COVID-19
Source: J Clin Invest. 2023 Jun 15;133(12):e170682. doi: 10.1172/JCI170682 (PMC10266785; doi:10.1172/JCI170682)
Supplement: Supplemental data 2 [file jci-133-170682-s092.pdf]

Investigator SCRIPT Pneumonia and Viral Episode Assessment and Outcome Evaluation

Study ID: _____________ Reviewer ○ 1 ○ 2 ○ 3 ○ CR: _______ Date

○ N/A – go to page 2	**Episode Category Assessment**
Is this for the initial pneumonia episode?<br><br>**Date of BAL in REDCap**: ______________	○ Yes, this is the initial pneumonia episode (implies > 1 episodes)<br>○ Yes, this is the initial and also final pneumonia episode<br>○ No, multiple episodes
If not initial, is this the final pneumonia episode?	○ Yes ○ No ○ N/A
Patient Category:<br>○ Clinical CAP (not hospitalized within the last 7 days)<br>○ Clinical HAP (current admission >48 hours or discharged from a healthcare facility within the last 7 days where admission >24 hours)<br>○ Clinical VAP (on ventilator >48 hours or reintubated < 24 hours from extubation)<br>○ Non-pneumonia control*<br>○ Infection [a]<br>○ Known Condition [b]<br>○ Unknown	*If non-pneumonia control, cause of infiltrate:<br>○ ARDS<br>○ Aspiration<br>○ Atelectasis<br>○ Fibrosis<br>○ Fluid overload<br>○ Heart failure/pulmonary edema<br>○ Pleural effusion<br>○ Pulmonary hemorrhage<br>○ Other: _______________<br>○ Unknown
[a] *If infection:*<br>○ Cholangitis/cholecystitis<br>○ Colitis<br>○ Other intra-abdominal<br>○ Line infection<br>○ Tracheobronchitis<br>○ Urinary tract<br>○ Wound/skin<br>○ Other: ___________________	[b] *If known condition, cause of fever/leukocytosis:*<br>○ Aspiration<br>○ Atelectasis<br>○ Drug fever<br>○ Pancreatitis<br>○ Other: ___________________<br>○ None<br>○ Unknown
**STOP here for non-pneumonia control**	
Choose one: (from Episode Category Assessment)<br>○ Viral* only<br>○ Bacterial/Viral* Co-Infection<br>○ Bacterial/Etiology defined<br>○ Culture-negative** (%PMNs ≥ 50%)<br>○ Culture-negative*** (%PMNs < 50%)<br>○ Indeterminate	******If viral, select virus type: (select all that apply)*<br>☐ Influenza<br>☐ SARS-CoV-2<br>☐ Other:___________
**Demographics page immune status is** ○ Immunocompromised ○ NON-Immunocompromised	
***If culture-negative (%PMNs ≥ 50%):*<br>○ Immunocompromised<br>○ Nonimmunocompromised	****If culture-negative (%PMNs < 50%):*<br>○ Neutropenic (ANC < 500/uL)<br>○ Immunocompromised<br>○ Nonimmunocompromised

**If Viral Only** ○ N/A	
Were appropriate antivirals administered?	○ Yes* ○ No ○ N/A – no appropriate antivirals
**If yes*, select antiviral: (select all that apply)*	☐ Influenza – oseltamivir<br>☐ Influenza-baloxavir<br>☐ Influenza-peramivir<br>☐ Influenza-other: ________________________<br>☐ SARS-CoV-2-remdesivir<br>☐ SARS-Cov-2-other: ________________________<br>☐ Adenovirus-cidofovir<br>☐ Herpes or Varicella-acyclovir<br>☐ RSV-ribavirin only if immunocompromised<br>☐ Other (please specify): ________________________
Were empirical antibiotics for this episode started prior to BAL?	○ Yes* ○ No
**If yes*, number of days **prior** to BAL collection?*	○ ≤ 48 hours before BAL<br>○ > 48 hours before BAL
Were empirical antibiotics **started/continued** after BAL collection?	○ Yes* ○ No
**If yes*, select number of days antibiotics were given:*	○ < 72 hours after BAL<br>○ 3-7 days after BAL<br>○ 8-10 days after BAL<br>○ 11-14 days after BAL<br>○ ≥ 15 days after BAL
**Viral Pneumonia Clinical Impression**	
Was viral retesting performed?	○ Yes*<br>○ No (go to Global Viral Clinical Impression)
**If yes*, PCR cleared?*	○ Yes by BAL<br>Date of 1st negative test with no subsequent positive: ________________<br>○ Yes by NP/OP (no BAL available or subsequently completed)<br>Date of 1st persistent negative test: ____________<br>○ Other viral procedure: __________________<br>Date of repeat test: ____________________<br>○ No<br>Date of last positive test: ________________
Global Viral Clinical Impression:<br>(*Go to PCT after*)	**Pneumonia (Check best answer)**<br>○ Cure<br>○ Persistence (repeat positive PCR until death, transfer to LTAC, needs time interval from last testing [i.e. positive within 7 days])<br>○ *Superinfection pneumonia (N/A if single episode only)*<br>○ Indeterminate (NP positive but BAL clear, no recent PCR prior to death or transfer to other facility)<br>**Other: (if applicable, all that apply)**<br>☐ Extrapulmonary infection (Single Viral) ***(see extra question)****

If ***extrapulmonary infection,** choose site (**check all that apply**) and corresponding documentation**:**

	**Documentation** ***(check most appropriate – only one)***			
**Site** ***(check all that apply)***	Definitive with positive culture	Definitive without culture proof	Radiologic/imaging only	Clinical suspicion only
☐ Cholangitis/cholecystitis	○	○	○	○
☐ Colitis /C. diff	○	○	○	○
☐ Other intra-abdominal (SBP, abcess, etc.)	○	○	○	○
☐ Line infection	○	○	○	○
☐ Tracheobronchitis	○	○	○	○
☐ Urinary Tract	○	○	○	○
☐ Wound/skin	○	○	○	○
☐ Empyema (unrelated to the index pna)	○	○	○	○
☐ Other (*please specify*): ____________________	○	○	○	○

**If Bacterial/Viral Co-Infection** ○ N/A (Viral Only)	
Were appropriate antivirals administered?	○ Yes* ○ No ○ N/A – no appropriate antivirals
**If yes*, select antiviral: (select all that apply)*	☐ Influenza – oseltamivir<br>☐ Influenza-baloxavir<br>☐ Influenza-peramivir<br>☐ Influenza-other: ________________________<br>☐ SARS-CoV-2-remdesivir<br>☐ SARS-CoV-2-other: ________________________<br>☐ Adenovirus-cidofovir<br>☐ Herpes or Varicella-acyclovir<br>☐ RSV-ribavirin only if immunocompromised<br>☐ Other (please specify): ________________________
Was viral retesting performed?	○ Yes*<br>○ No (go to *If initial sample, has the patient…*)
**If yes*, PCR cleared?*	○ Yes by BAL<br>Date of 1st negative test with no subsequent positive: _______________<br>○ Yes by NP/OP (no BAL available or subsequently completed)<br>Date of 1st persistent negative test: ___________<br>○ Other viral procedure: _________________<br>Date of repeat test: ___________________<br>○ No<br>Date of last positive test: _______________

Continue If Bacterial/Viral Co-Infection or Complete If Bacterial Only ○ N/A (Viral only)	
If initial sample, has the patient been actively treated for pneumonia for **more** than 24 hours prior to sample collection?	○ Yes* ○ No ○ N/A<br><br>**BAL Collection Date/Time:** ______________________<br>*(Obtain from EPIC - Microbiology)*
**Starting antibiotics for pneumonia:** _____________<br><br>**Start date for antibiotics for pneumonia:** _____________<br><br>**If* ***yes***, *how many calendar days has patient been treated? (Day BAL – Day abx started)*	__________________days ○ N/A
Appropriate initial antibiotics?<br>*(in the 72 hrs. preceding culture results)*<br><br>**Refer to biofire results if applicable*	○ Yes ○ No ○ N/A – no bacterial pathogen
Were antibiotics for pneumonia discontinued on D 7-8?	○ Yes*<br>○ No<br>○ N/A**(death/transfer to another institution)<br><br>**if* ***Yes***, *finish following clinical impression and go to PCT*<br>***If* ***N/A***, *skip "clinical impression" questions and go to PCT*
**Clinical Impression**	
Clinical Impression at **D7-8**:<br><br>**must be actively treated at this time point*	**Pneumonia (Check best answers)**<br>○ Cure<br>☐ Persistence *(check all that apply)*<br>☐ Positive Culture<br>☐ Positive PCR<br>☐ Abscess / Cavity<br>☐ Empyema<br>☐ Endocarditis<br>☐ Other: ________________________<br>☐ Superinfection pneumonia<br>☐ Indeterminate<br><br>**Other: (if applicable, all that apply)**<br>☐ Extrapulmonary infection ***(see extra question)****<br>☐ Prophylaxis only

If ***extrapulmonary infection,** choose site (**check all that apply**) and corresponding documentation**:**

	**Documentation** *(check most appropriate – only one)*			
**Site** ***(check all that apply)***	Definitive with positive culture	Definitive without culture proof	Radiologic/imaging only	Clinical suspicion only
☐ Cholangitis/cholecystitis	○	○	○	○
☐ Colitis /C. diff	○	○	○	○
☐ Other intra-abdominal (SBP, abcess, etc.)	○	○	○	○
☐ Line infection	○	○	○	○
☐ Tracheobronchitis	○	○	○	○
☐ Urinary Tract	○	○	○	○
☐ Wound/skin	○	○	○	○
☐ Empyema (unrelated to the index pna)	○	○	○	○
☐ Other (*please specify*): ____________________	○	○	○	○

Were antibiotics for pneumonia discontinued on D10?	○ Yes*<br>○ No<br>○ N/A**(death/transfer to another institution)<br><br>**if **Yes**, finish following clinical impression and go to PCT*<br>***If **N/A**, skip "clinical impression" questions and go to PCT*
Clinical Impression at **<u>D10</u>**:<br><br>**must be actively treated at this time point*	**Pneumonia (Check best answers)**<br>○ Cure<br>☐ Persistence *(check all that apply)*<br>☐ Positive Culture<br>☐ Positive PCR<br>☐ Abscess / Cavity<br>☐ Empyema<br>☐ Endocarditis<br>☐ Other: ______________________<br>☐ Superinfection pneumonia<br>☐ Indeterminate<br><br>**Other: (if applicable, all that apply)**<br>☐ Extrapulmonary infection *(**see extra question**)**<br>☐ Prophylaxis only

If ***extrapulmonary infection,** choose site (**check all that apply**) and corresponding documentation**:**

**Site** ***(check all that apply)***	**Documentation** ***(check most appropriate – only one)*** Definitive with positive culture	Definitive without culture proof	Radiologic/imaging only	Clinical suspicion only
☐ Cholangitis/cholecystitis	○	○	○	○
☐ Colitis /C. diff	○	○	○	○
☐ Other intra-abdominal (SBP, abcess, etc.)	○	○	○	○
☐ Line infection	○	○	○	○
☐ Tracheobronchitis	○	○	○	○
☐ Urinary Tract	○	○	○	○
☐ Wound/skin	○	○	○	○
☐ Empyema (unrelated to the index pna)	○	○	○	○
☐ Other (*please specify*): ____________________	○	○	○	○

Were antibiotics for pneumonia discontinued on D14?	○ Yes*<br>○ No<br>○ N/A**(death/transfer to another institution)<br><br>**if **Yes**, finish following clinical impression and go to PCT*<br>***If **N/A**, skip "clinical impression" questions and go to PCT*
*If **no**, what date were antibiotics for pneumonia stopped?*	Stop date for antibiotics for PNA: ____________<br>○ Stop date unknown (patient was discharged on antibiotics for pneumonia)
Clinical Impression at **D14**:<br><br>**must be actively treated at this time point*	**Pneumonia (Check best answers)**<br>○ Cure<br>☐ Persistence *(check all that apply)*<br>☐ Positive Culture<br>☐ Positive PCR<br>☐ Abscess / Cavity<br>☐ Empyema<br>☐ Endocarditis<br>☐ Other: ______________________<br>☐ Superinfection pneumonia<br>☐ Indeterminate<br><br>**Other: (if applicable, all that apply)**<br>☐ Extrapulmonary infection ***(see extra question)****<br>☐ Prophylaxis only

If ***extrapulmonary infection,** choose site (**check all that apply**) and corresponding documentation**:**

**Site** ***(check all that apply)***	**Documentation** ***(check most appropriate – only one)***			
	Definitive with positive culture	Definitive without culture proof	Radiologic/imaging only	Clinical suspicion only
☐ Cholangitis/cholecystitis	○	○	○	○
☐ Colitis /C. diff	○	○	○	○
☐ Other intra-abdominal (SBP, abcess, etc.)	○	○	○	○
☐ Line infection	○	○	○	○
☐ Tracheobronchitis	○	○	○	○
☐ Urinary Tract	○	○	○	○
☐ Wound/skin	○	○	○	○
☐ Empyema (unrelated to the index pna)	○	○	○	○
☐ Other (*please specify*): ____________________	○	○	○	○

PROCALCITONIN: (within 72 hrs of BAL/NBBAL Collection) [All categories]

Are serial PCTs available?<br>*Note: minimum of 2 measurements within this treatment episode*	○ Yes* ○ No
**If yes**, does the patient meet PCT criteria for cure?*	○ Yes, (check criteria below) ○ No<br>☐ 80% change/decrease from baseline<br>☐ Absolute value of $\leq$ 0.5 ng/mL

IF FINAL EPISODE: [All categories]

Overall Global Clinical Cure **please refer to guidelines*

Overall Success?

○ **Yes**

→ If Yes, all criteria? (see text box below)

○ Yes

○ No

→ If No, was patient extubated **before** day 7?

○ Yes

○ No

1. survive duration of treatment
2. able to stop antibiotics for pneumonia
   a. Continuation of same antibiotics > 14 days but signs/symptoms of pneumonia stable (WBC, secretions, oxygenation, radiograph) or improving?
3. causative pathogen disappears from respiratory secretions or no further samples
   a. %PMNs in repeat BAL < 50% in non-neutropenic
4. clinical manifestations of pneumonia improve/resolve (e.g., fever, secretions, WBC, hypoxemia, septic shock)
5. ability to wean from vent or at least initiate SBTs
   a. Drop in minute ventilation and/or improvement in oxygenation
   b. Patient returned to pre-pneumonia ventilator/ECMO status or weaning from vent

○ **No**

→ Cause of failure:*

☐ Antibiotics for another indication in addition to suspected/proven PNA

☐ Persistence *(check all that apply)*

☐ Positive Culture

☐ Positive PCR

☐ Abscess / Cavity

☐ Empyema

☐ Endocarditis

☐ Other: ________________

☐ Persistent inflammation only

☐ Recurrence

☐ Superinfection pneumonia

** Check best answers*
